# Children's Mental Models of Prenatal Development

**DOI:** 10.3389/fpsyg.2018.01835

**Published:** 2018-10-01

**Authors:** Tessa J. P. van Schijndel, Sara E. van Es, Rooske K. Franse, Bianca M. C. W. van Bers, Maartje E. J. Raijmakers

**Affiliations:** ^1^Research Institute of Child Development and Education, University of Amsterdam, Amsterdam, Netherlands; ^2^Arkin NPI Centre for Personality Pathology in Amsterdam, Amsterdam, Netherlands; ^3^Developmental Psychology, Department of Psychology, University of Amsterdam, Amsterdam, Netherlands; ^4^NEMO Science Museum, Amsterdam, Netherlands; ^5^Educational Studies, Faculty of Behavioral and Movement Sciences, Free University Amsterdam, Amsterdam, Netherlands

**Keywords:** prenatal development, mental models, naïve biology, fragmented knowledge, representational-redescription, latent variable models

## Abstract

Children's thinking about prenatal development requires reasoning about change that cannot be observed directly. How do children gain knowledge about this topic? Do children have mental models or is their knowledge fragmented? In Experiment 1, results of a forced-choice questionnaire about prenatal development (6- to 13-year-olds; *N* = 317) indicated that children do have a variety of coherent, grade-related, theories about early shape of the fetus, but not about bodily functions. Coherence of the mental models was enhanced by a preceding generative task. Children's mental models were in agreement with reasoning about natural transformations (Rosengren et al., 1991) and constraints in representational flexibility (Karmiloff-Smith, 1992). In Experiment 2, an open-question interview was administered (6- to 12-year-old children; *N* = 38). The interview resulted in grade-unrelated, incoherent responses. This study contributes to a deeper understanding of naïve biology and to the effects of different methodologies being used in the area of mental models.

## Introduction

Children gain a great deal of knowledge about the world around them during childhood (Carey, 1985). For example, children experience gravity with their body, observe objects falling down, are told about gravity as a force, and see movies about weightless astronauts in space. To integrate some of this knowledge into a coherent, not necessarily scientifically correct, concept of gravity is an extremely difficult task. The possible sources of knowledge vary between topics (such as gravity, floating and sinking, the shape of the earth) and between children, depending on age and culture (e.g., Springer, 1999; Harris and Koenig, 2006; Gelman, 2009). A fundamental question regarding each knowledge domain is whether children form theory-like, coherent and internally consistent ideas, i.e., mental models (Vosniadou and Brewer, 1992; Straatemeier et al., 2008), or whether their knowledge about the topic remains fragmented until they learn a scientific concept (Vosniadou et al., 2004; diSessa, 2008). That is, following Straatemeier et al. we take as a defining characteristic of a mental model a coherent and internally consistent idea, leading to a coherent pattern of responses to a relevant set of questions. To gain a better understanding of knowledge development in general, it is important to gain a better understanding of knowledge development for specific domains.

The aim of the present study is to examine the development of children's knowledge in the domain of biology, in particular prenatal development. Children may have multiple sources of knowledge about prenatal development, such as facts that are being told, movies, pictures in a pregnancy book, or knowledge about the development of other living things including themselves. In naïve biology, most researchers state that children develop some coherent ideas, although they may disagree about the origin of these ideas. Some researchers link the early formation of biological theories to innate or early acquired predispositions or cognitive biases (Keil, 1992; Mandler and McDonough, 1993; Gelman and Hirschfeld, 1999; Kelemen, 1999). Other researchers emphasize the importance of experience (Carey, 1985; Hatano and Inagaki, 1994; Inagaki, 1997; Au and Romo, 1999; Springer, 1999) and state that the main principle for theory formation is the acquisition of factual knowledge combined with key inferences from this knowledge. A prenatal baby is not directly observable, but children could make inferences about a prenatal baby on basis of their factual and experience-based knowledge of a post-natal baby. Hence, studying children's knowledge of prenatal development provides not only insight in naïve biology but reveals also aspects of children's representational flexibility.

### Naive knowledge of prenatal development

The literature on children's naïve ideas about prenatal development is scarce. Most studies focus on either conception (e.g., Bernstein and Cowan, 1975), inheritance (Springer and Keil, 1989), or birth models (e.g., Nagy, 1953; Kreitler and Kreitler, 1966). Discussing the creation of babies, Kreitler and Kreitler (1966) argued that 4- and 5-year-olds' views are based on three theories: (1) the baby is created in the mother's belly from the food she eats, (2) the baby has always existed in the mother's belly, or (3) the baby is swallowed by the mother. The specific topic of prenatal development has rarely been focused on, however. Zoldosova and Prokop (2007) performed a qualitative analysis of drawings and structured interviews based on a relatively small group of participants. They concluded that four broad categories existed in children's drawings about prenatal development: (1) drawings of a fetus without indicating development, (2) drawings of a pregnant woman with a fetus in her belly, but without indicating development, (3) drawings of a pregnant woman with a developing fetus, and (4) drawings of pregnancy development. Furthermore, they found that children showed large variation in the body parts they drew. For example, 70% of the children drew an umbilical cord. From the interviews only qualitative observations were reported, e.g., that children had very limited knowledge about fetuses' insides, although the older children mentioned the umbilical cord in the context of food uptake.

Two additional topics in naïve biology are of particular interest for the present study: children's understanding of biological growth and transformations, and children's understanding of bodily functions. Preschool-age children already have naïve ideas about biological growth (Carey, 1985; Inagaki and Hatano, 2004; Gottfried and Gelman, 2005). Using a forced-choice experimental paradigm, Rosengren et al. (1991; French et al., 2018) showed that preschoolers' ideas about natural transformations are coherent. They understand that animals get larger and not smaller with age, but only older children and adults accept rather dramatic changes of color and shape. Reasoning about human insides is a larger area of research (e.g., Gellert, 1962; Carey, 1985), which concerns the type of explanations children give for bodily phenomena (Miller and Bartsch, 1997; Hatano and Inagaki, 1999; Inagaki and Hatano, 2004). To reason about changes in bodily functions during prenatal development, children should understand that some functions are important for staying alive. Several studies (Gellert, 1962; Jaakkola, 1997) show, by using interviews, that preschoolers know that humans need to breathe air, to eat, and to drink to stay alive. However, young children were shown to have very little knowledge about human insides, such as the lungs and digestive system. A large part of the knowledge development about the mechanistic causality of people's insides takes place in later childhood until adulthood (Morris et al., 2000).

One way of reasoning about unknown aspects of prenatal development is to project known properties from other living things, that is, from post-natal humans, animals, or plants. Hatano and Inagaki (1994; Inagaki and Hatano, 1996, 2004) conclude that at 5 years of age most children have an integrated category for living things. Children reported that growing, taking food/water, and aging/dying are similar phenomena for animals and plants.

### Representational redescription

It can be concluded that children have some factual knowledge about a newborn, about bodily shape and bodily functions, such as eating, drinking, and breathing. Hence, to reason about prenatal bodily shape and bodily functions the child could transform this knowledge. Children's ability to transform its conceptual knowledge is believed to be dependent on the flexibility of their internal representations. In her representational redescription (RR) theory Karmiloff-Smith (1990) describes the acquisition of knowledge through sequential phases, starting with a representation of knowledge in a procedural, implicit way, followed by re-representations at different levels of abstraction. According to RR theory, representational flexibility is dependent on the level of representation. This theory has found support in several areas of cognitive development, such as language, mathematics, and physics (Karmiloff-Smith, 1992; Hollis and Low, 2005; Critten et al., 2007). The sequence of phases is found to be domain general, but the individual level of representational flexibility is believed to differ between specific topics.

Karmiloff-Smith (1990; Spensley and Taylor, 1999) used children's drawings to explore representational flexibility. She focused on the constraints children have in their representational flexibility by looking at what changes children make in their concept representation (e.g., a house or human being) when asked to draw a non-existing concept (e.g., a fake house or human being). Karmiloff-Smith's results of the “non-existing human being” task, show that changes introduced by preschoolers mainly involved deletions and changes in size and shape, whereas older children often changed position and orientation of elements and added elements from other conceptual categories.

Based on former research in naïve biology and taking into consideration RR theory (Karmiloff-Smith, 1990), we predict children to have the following mental models of prenatal development. I) A *no change* model, predicting that a fetus has the same shape and equivalent bodily functions as a newborn. II) A *growth* model, predicting growth of the bodily shape and gradual increase of bodily functions. What children observe in post-natal development is that the shape grows and that bodily functions stay equal. Both constant and increasing bodily functions would agree with children having limited representational flexibility but have the understanding of *growth*. III) An alternative naïve idea could be the *outgrowth* of body elements and the *emergence* of functions, analogous to a growing plant with outgrowing branches. Some children might use the analogy of how a plant grows new branches. Such a transformation that consists of the addition of elements agrees with a higher level in RR. IV) A child in an even later representational stage of its concept of a newborn would allow for more dramatic changes. It would accept that the shape of a fetal hand looks *different* from that of a newborn's hand and that some bodily functions are implemented differently.

### Mental models vs. fragmental knowledge

Most authors claim that children have coherent ideas about biological topics, but this claim has not often been tested experimentally (but see e.g., Rosengren et al., 1991). The question of whether children's knowledge about specific topics is coherent and represented in mental models or whether it is fragmented has been a topic of heated debate (Vosniadou et al., 2004; diSessa, 2008). Theorists on the coherence side of this debate maintain that children's naïve ideas are organized into coherent and consistent theories, which structure everyday thinking and are more or less resistant to change (Johnson-Laird, 1983; Wellman and Gelman, 1998). Different knowledge domains have been studied within this approach: the shape of the earth (Vosniadou and Brewer, 1992); the motions of the earth, sun, and moon; the relative location of the earth, sun, and moon in space and the day-night cycle (Samarapungavan et al., 1996); and, evolution and speciation (Samarapungavan and Wiers, 1997). A more specific issue that is central in the debate is whether children form mental models “on the spot.” According to Vosniadou et al. (2004) children form on-the-spot dynamic situation-specific representations for the specific purpose of answering questions that are being posed to them. They argue that tasks like drawing, making clay models or responding to open-ended questions encourage children to make generative use of the scientific information that they have at their disposal, which encourage on-the-spot formation of mental models, thereby increasing the coherence of the responses.

In contrast to the coherence theorists, researchers on the other side of the debate claim that children's naïve knowledge is fragmented. diSessa (1988), for instance, claims that intuitive physics stems from fragmented knowledge, represented like a set of loosely connected ideas. In line with this view, some researchers have concluded that children's knowledge of the earth is fragmented (Nobes et al., 2003; Straatemeier et al., 2008).

### The measurement debate

A complicated aspect of this discussion is that differences in conclusions are related to the used experimental methodologies. Vosniadou et al. (2004) argued that the best way to investigate children's knowledge is by making use of generative methods, such as drawing. Several points of criticism exist concerning the use of drawings in the study of mental models. Firstly, the constraints on the planning of drawings may hamper children in divulging mental models (Nobes et al., 2005). Secondly, it is difficult to establish objective scoring methods (Brainerd, 1973; in Straatemeier et al., 2008) although, results of drawings can be valuable when acceptable levels of inter-rater reliability are maintained. Thirdly, unexpected mental models cannot be found by using fixed scoring schemes. Finally, when aiming to test the existence of mental models, rather than fragmented knowledge, a measure of coherence is needed. Coherence can only be measured when multiple responses are scored, such as in a questionnaire or in an interview, but not a single drawing.

Forced-choice questionnaires have been one of the methods of choice within the area of mental models of the earth (Nobes et al., 2003; Straatemeier et al., 2008; Frède et al., 2011), leading to the conclusion that children do not have mental models of the shape of the earth. Vosniadou et al. (2004) criticized this method for several reasons. Firstly, responses to the forced-choice questionnaire may be biased because the choice of response options is limited. This problem can be overcome if the construction of the test is based on extensive piloting on possible alternative models. Secondly, because children only need to recognize correct information, they might perform better on forced-choice questionnaires. A solution to this problem could be the use of an additional open-ended interview.

The use of open-ended interviews is not without problems, either. In order to arrive at a full understanding of the underlying conceptual structures, similar questions addressing the same issues are asked, resulting in a prolonged method of repeated questioning. This unnatural situation may confuse children, because everyday conversational rules do not apply (Siegal, 1997). Hence, the structure of the interview should allow the collection of sufficient responses with only few follow-up questions. Another criticism Siegal emphasizes in the use of interviews is children's limited vocabulary, partially unknown to the researcher. The use a model may facilitate the understanding of the child.

To conclude, the most favorable methodology to examine the existence of mental models of prenatal development is either a forced-choice questionnaire in which all perceptible mental models are represented or an interview set up in such a way that conversational rules apply and a model is used to facilitate the child's understanding of the question. With the former method coherence can be measured best because we can apply a larger number of comparable items and we can study a larger number of children. In the current study, both methodologies were used and compared. In experiment 1 a questionnaire was designed in which pictures were used instead of written response categories, to avoid problems related to verbal methodologies. In experiment 2, in addition to the forced-choice questionnaire, an interview was administered. In the questionnaire and the interview similar questions were asked. We examined whether children demonstrated the same mental models in the interview as in the questionnaire. In addition, a drawing task on prenatal development was included in the design of both experiments. A first purpose of administering the drawing task was to examine the effect of a preceding generative task on the coherence of mental models. Moreover, by assigning the drawings to a mental model according to well-described scoring rules, we studied the relation between the results of the three assessments methodologies.

## Experiment 1

### Method

#### Participants

We tested 317 children (age: *M* = 9.35, *SD* = 1.84) from three primary schools providing regular education in different parts of the Netherlands. The Review Board of the Faculty of the Social and Behavioral Sciences of the University of Amsterdam reviewed and approved all consent and study procedures. An opt-out consent procedure was used for children in two of the three schools. Parents received written letters by regular mail with a reply sheet that could be given to the teacher in case the child was not allowed to participate or they could e-mail or phone the researcher or the ethics board. On request of the third school a written active consent procedure was used. Parents received written letters with a reply sheet that could be given to the teacher in case the child was allowed to participate. The sample consisted of children between 6 and 12 years old: participants included 43 children from Grade 1, age 6–7 (*M* = 6.56, *SD* = 0.59); 50 children from Grade 2, age 7–8 (*M* = 7.74, *SD* = 0.60); 68 children from Grade 3, age 8–9 (*M* = 8.75, *SD* = 0.63); 47 children from Grade 4, age 9–10 (*M* = 9.91, *SD* = 0.69); 51 children from Grade 5, age 10–11 (*M* = 10.84, *SD* = 0.51); and 58 children from Grade 6, age 11–12 (*M* = 11.76, *SD* = 0.60).

#### Materials

##### Questionnaire

The questionnaire was designed for children aged six and older, following Siegler's (1981) rule assessment methodology (RAM). It was expected that a child with a specific mental model would generate a specific response pattern for a set of items. Items were constructed such that maximum differentiation was possible between the expected mental models: (1) the *no-change* model, (2) the *growth* model, (3) the *outgrowth* model, and (4) the *different* model. The themes of the questions used in the questionnaire were based on children's misconceptions of prenatal development reported in earlier studies (Gellert, 1962; Jaakkola, 1997; Zoldosova and Prokop, 2007), and in discussions and drawings at a science education workshop^1^.

**Figure 1:**
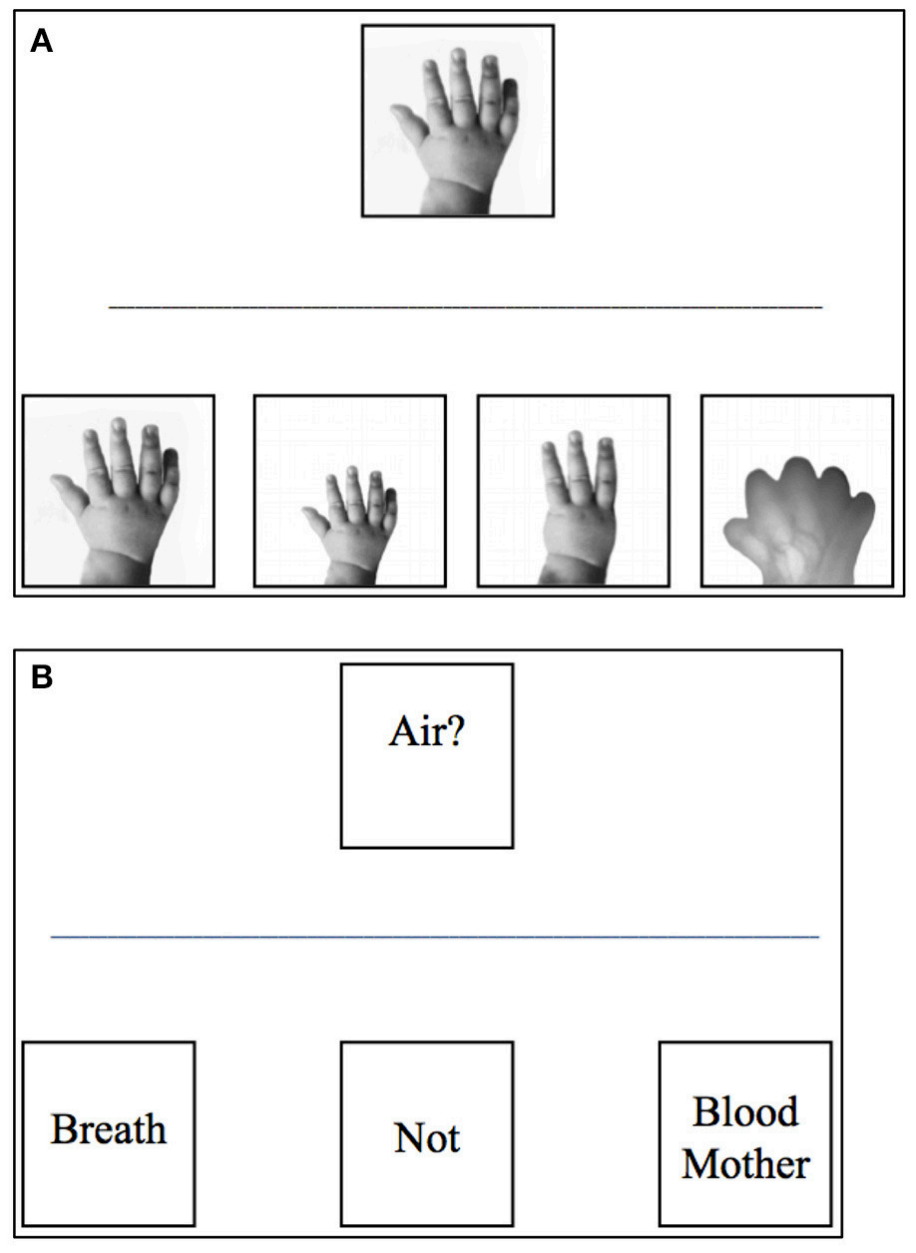
Examples of items in the questionnaire. **(A)** Shape item about the hand. **(B)** Function item about breathing. Of each item the upper figure depicts the shape (function) of a newborn; the lower figures are the four response options for shape items (no-change, growth, outgrowth, and different) or three response options for function items (different, no-change, absence). In the questionnaire the response options were presented in random order. Note: **B**) is a translation of the original Dutch item.

The questionnaire started with a trivial example item, followed by 15 questions on prenatal development. These questions covered an early (part 1) and a late stage of prenatal development (part 2). In turn, the items within each part concerned shape of the body or bodily functions of the fetus. Topics covered in the shape-related items were the hand, leg, ear, foot, arm, and eye. There were 6 early shape (ES) and 3 late-shape items (LS: hand, leg, ear). Topics covered in the function-related items were breathing, drinking and maintaining body temperature. There were 3 early function (EF) and 3 late-function items (LF). In the booklet the response options of the shape-related items were depicted with adapted photographs. The response options of the function-related items were given in a few short words. The order of the response options was randomized. Examples of 2 items, an ES and a LF item, are depicted in Figure 1. The experimenter presented the ES item in front of the classroom as follows: *This is what the hand of a baby looks like, just after being born. Which picture resembles the hand of a baby most, when the baby still has to stay in the belly of the mother for a long, long time?* The experimenter presented the LF item in front of the classroom as follows: *After being born a baby needs to breathe air to stay alive. How does the baby get air just before being born?* The complete questionnaire is available as Supplementary Material.

##### Drawing task

Participants were asked to make two drawings of a fetus: one in an early stage and one in a late stage of development. The assignment for the first drawing was: “draw a baby in the belly of the mother, for the case when the baby has to stay in the belly of the mother for a long, long time.” The assignment for the second drawing was: “draw a baby in the belly of the mother, right before it is born.” Children received a format on A3-sized paper depicting three pictures of a mother, the first two with an empty square on the mother's belly where the baby had to be drawn, and the third depicting a mother with her newborn.

The drawings were scored based on the types of changes that appeared between the first and the second drawing. Types of scored change were: growth from early to late stage of fetal development, deletion in the early stage as compared to the late stage, and changes in shape from early to late stage. Drawings were sorted into four categories related to the predicted mental models; (1) *no-chang*e, (2) *growth*, (3) *outgrowth*, and (4) *different*. The type of change between the drawings representing the highest level of conceptual change was used as a criterion for categorizing the drawing. For example, if both growth and a different shape were apparent in a drawing, the drawing was classified in category 4.

#### Design and procedure

The experimental procedure consisted of two parts: the questionnaire and the drawing task. These two tasks were administered group wise in a classroom setting. Children were tested under two conditions, counterbalancing the order of the questionnaire and the drawing. The instructions of all tasks, including the questionnaire items, were read out loud by the test leader. Additionally, an enlarged version of all questions was projected on a screen. The tasks took about 20 min each, after which children could ask questions.

#### Statistical analyses

The forced-choice questionnaires were analyzed to detect mental models of prenatal development. Although we formulated clear expectations about the mental models we apply explorative Latent Class Analysis (LCA; McCutcheon, 1987; Visser, 2011) to allow the detection of unexpected mental models or the absence of mental models. LCA provides a statistically reliable method to detect rules (Jansen and van der Maas, 1997; Van Der Maas and Straatemeier, 2008) or mental models (i.e., Straatemeier et al., 2008) from non-verbal responses. LCA is a statistical technique for models with categorical manifest variables (forced-choice questionnaire responses) and a categorical latent variable (the latent classes). In our study a latent class can be interpreted as a response pattern related to a specific mental model. A latent class model is defined by unconditional probabilities and conditional probabilities, which are estimated from the data. Unconditional probabilities define the probability of belonging to a latent class, i.e., the size of the class. Conditional probabilities represent the probability of each answer option, given that the subject belongs to a specific class. To calculate maximum likelihood estimates of the parameters, we used the DepmixS4 package (Visser and Speekenbrink, 2010) for the R program for statistical computing (R Development Core Team, 2010). Model selection criteria, especially the Bayesian Information Criterion (BIC; Schwarz, 1978), were used to choose the optimal, most parsimonious model, i.e., the optimal number of classes that described the data. The model with the lowest BIC was considered to be the most parsimonious, best fitting model. If children have a mental model of prenatal development, we should find a limited number of latent classes with an interpretable pattern of conditional probabilities. Because response patterns of classes do not need to be formulated beforehand, it is possible to detect alternative mental models. Obviously, the design of the questionnaire restricted the models we could find, but this is inherent to all closed-form assessment tools.

### Results

#### General

The internal consistency of the entire questionnaire as expressed by Cronbach's Alpha was 0.82 with the response options taken as an ordinal variable (*no-change, growth, outgrowth, different*). Non-parametric (Spearman's rho) correlations between items varied between 0.49 and 0.79 for the ES items, between 0.05 and 0.18 for the EF items, between 0.90 and 0.94 for the LS items, and were 0.01 and 0.20 for the LF items. The low correlations between function items will be addressed in the next section. Table 1 shows the distributions of responses. For the ES items, the frequency of the *no-change* response was very low. Hence, in further analysis the *no-change* response was combined with the *growth* response for the ES items. We model the three LS items separately.

**Table 1:** Distributions of responses for the questionnaire items in Experiment 1.

**Item**	**No-change**	**Growth**	**Outgrowth**	**Different**
**EARLY SHAPE**				
Hand	2.5	36.6	2.8	58.0
Leg	1.9	39.6	13.0	45.6
Ear	0.3	46.4	26.8	26.5
Foot	0.3	49.5	2.8	47.3
Arm	0	37.9	11.0	51.1
Eye	0.9	36.0	14.5	48.6
**EARLY FUNCTION**				
Drinking	2.2		1.6	96.2
Keeping Warm	0.3		3.2	96.5
Breathing	15.5		11.0	73.5
**LATE SHAPE**				
Hand	52.1	46.7	0.9	0.3
Leg	51.4	47.6	0.6	0.3
Ear	51.3	48.1	0	0.6
**LATE FUNCTION**				
Drinking	6.3		1.9	91.8
Keeping Warm	1.3		1.9	96.8
Breathing	15.5		9.5	75.1

*Frequencies are given as percentages of children (N = 317)*.

The Function items (EF and LF) showed little variance in responses and there was no striking difference in frequencies between EF and LF. Correlations between items on EF and LF were between 0.67 and −0.14. We modeled the six function items together in a separate latent class analysis to observe whether children responded coherently between the early and late stage. For the latent class analyses, the data of the two task-sequence conditions were taken together.

#### Mental models of bodily shape

**Figure 2:**
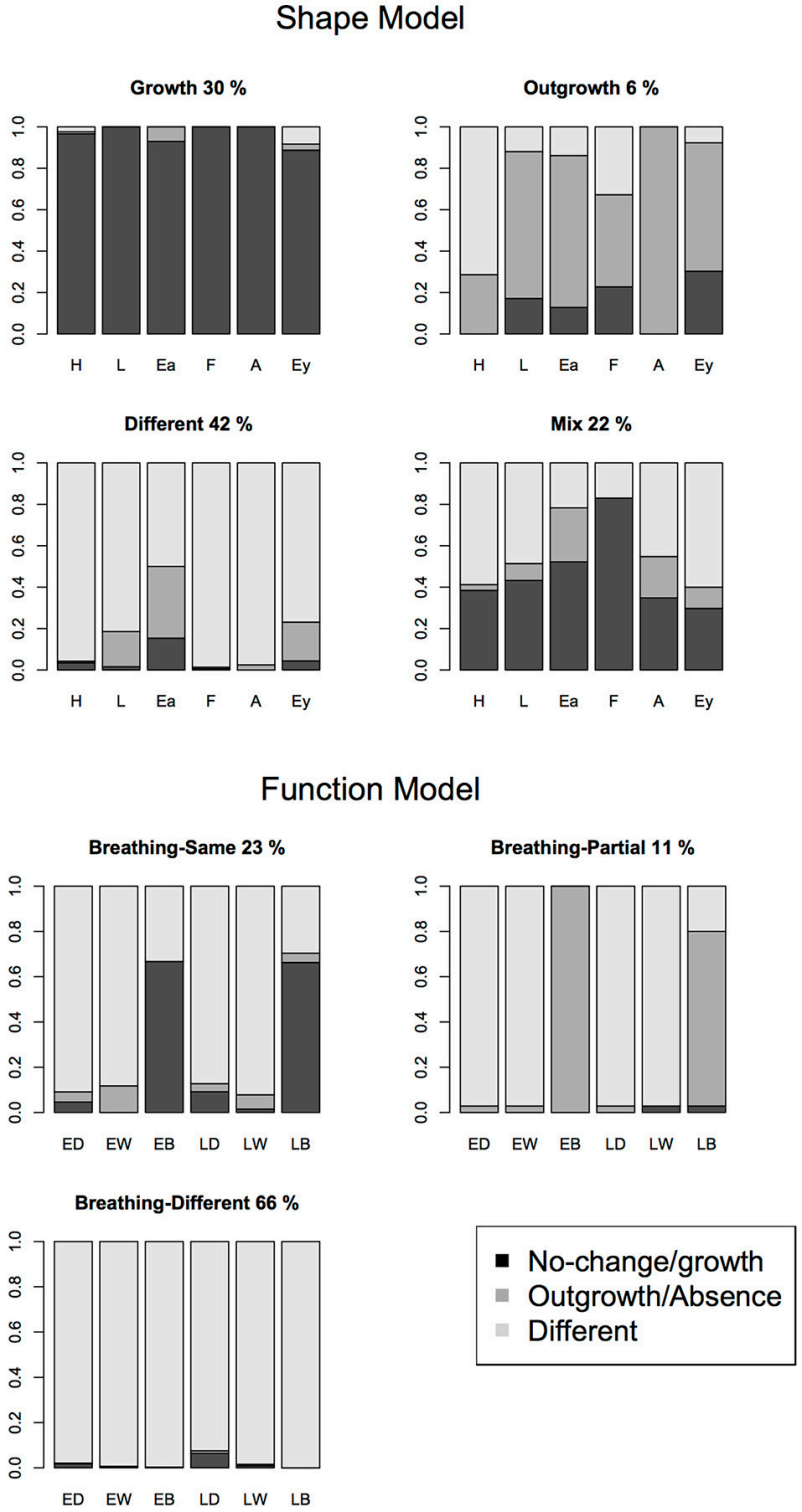
Graphical depiction of the latent class models of early shape items (upper part) and early and late function items (lower part). Each figure shows the conditional response probabilities for all items in one of the latent classes. Black refers to the conditional probability of the *no-change/growth* response, dark gray refers to the *outgrowth* response, and light gray refers to the *different* response. For the shape model the respective items are Hand (H), Leg (L), Ear (Ea), Foot (F), Arm (A), and Eye (Ey). For the function model the respective items are Early Drinking (ED), Early Warm (EW), Early Breathing (EB), Late Drinking (LD), Late Warm (LW), Late Breathing (LB).

To examine whether children showed mental models of bodily shape in the early stage, latent class analysis was first performed on the responses of the ES items. Table 2 (upper section) shows the fit statistics of the explorative latent class models with increasing number of classes. Based on the BIC, a four-class model yielded the most parsimonious, best-fitting model. The response patterns per class are depicted in Figure 2. Thirty percent of the children chose the *growth* response for all items, implying a growth-only mental model of prenatal development. For example, on the early shape leg (ES L) item, these children tended to choose the smaller version of the complete leg. Forty-two percent of the children chose the *different* responses for most items, implying a mental model that includes important changes of shape. For example, on the early shape leg (ES L) item, these children tended to choose the different looking fetal leg. Six percent of the children chose the *outgrowth* response option for most items, although less so for the hand and the foot, implying a mental model that allows for outgrowth of body parts. For example, on the early shape leg (ES L) item, these children tended to choose the partially complete leg (leg until knee). Although the responses of only a small subgroup of children was best described by this class, it does contribute to the most parsimonious best-fitting description of the data. Finally, 22% of the children chose a mixed set of responses, *growth* and *different* response in an unsystematic combination.

**Table 2:** Fit statistics for latent class models.

**Model**	**# classes**	**LogL**	**df**	**BIC**
Early shape	2 class	−1300.8	25	2745.5
	3 class	−1242.1	38	2703.0
	4 class*	−1201.7	51	2697.0
	5 class	−1184.9	61	2738.1
Late shape	1 class	−704.8	8	1455.6
	2 class*	−357.6	17	813.1
	3 class	−343.9	26	837.4
Function 6 items	1 class	−735.4	12	1539.9
	2 class	−648.2	25	1440.2
	3 class*	−609.0	38	1436.7
	4 class	−592.4	51	1478.4

* *Indicates the most parsimonious, best fitting model; LogL, Loglikelihood; df, degrees of freedom; BIC, Bayesian Information Criterion; no restrictions were added*.

*N = 316*.

A second series of models were fit to the responses of the LS items. Table 2 (second section) shows the fit statistics. Based on the BIC, a two-class model was selected as the most parsimonious, best-fitting model. Fifty-one % of the children chose the *no-change* response for all items (estimated conditional parameters: 0.99, 0.99, 0.98 for the hand, leg ear items). Forty-nine percent of the children chose the *growth* response for all items (estimated conditional parameters: 0.95, 0.96, 0.95 for the hand, leg ear items).

#### Mental models of bodily functions

To examine whether children showed mental models for bodily functions, latent class analysis was performed on the responses of the function items: 3 EF items and 3 LF items. Table 2 (third section) shows the fit statistics of the explorative latent class models with increasing number of classes. Based on the BIC, the three-class model was selected as the most parsimonious, best-fitting model. The response patterns per class are depicted in Figure 2, lower panels. Twenty-three percent of the children chose a *no-change* response for the breathing items, combined with a *different* response for the other items. Eleven percent of the children chose the *absence* response for the breathing items, again combined with a *different* response for the other items. Sixty-six percent of the children chose a *different* response for all items. This implies that children systematically chose responses other than *different* only for the breathing items.

#### Grade related differences

**Figure 3:**
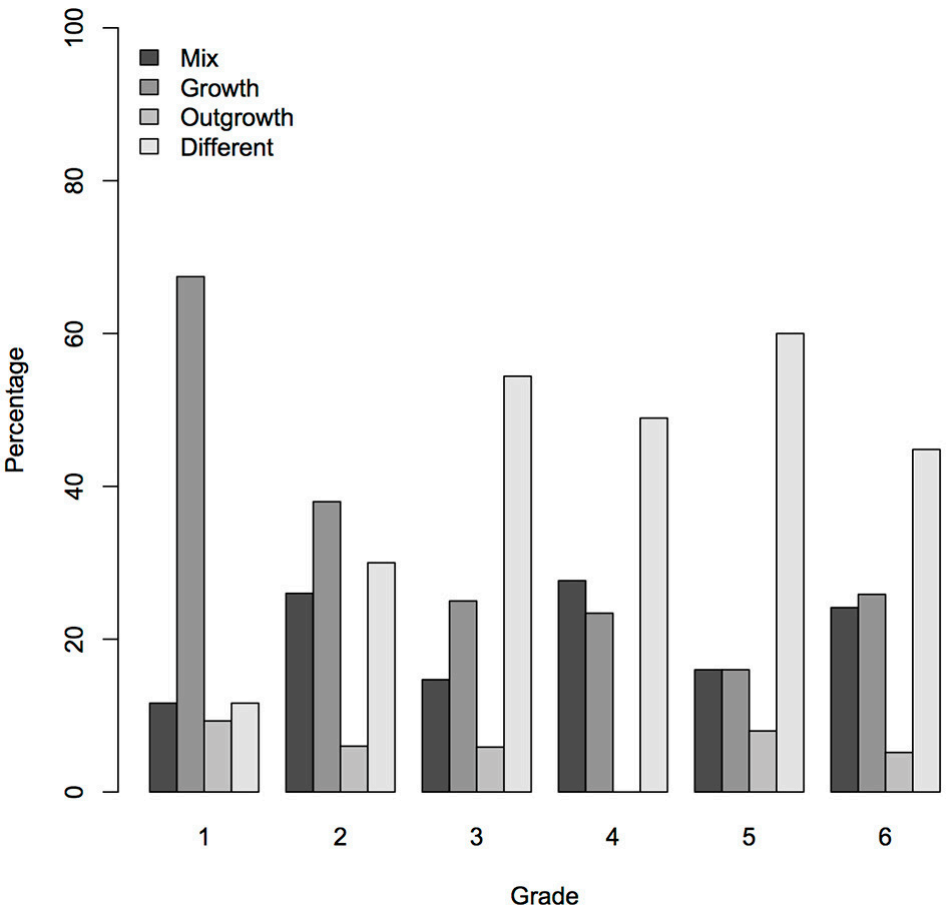
Graphical depiction of the relation between grade and mental model based on the early shape items. For each grade the distribution of the children over mental models is shown (in percentages).

For the Shape model and the Function model, participants' were assigned to a latent class based on the posterior probabilities of their responses, given the model. For the Shape model, the Wald criterion demonstrated that grade made a significant contribution to predicting class membership (*z* = 4.45, *p* < 0.001). Figure 3 shows that the *growth* class is most frequent for grade 1, whereas the *different* class is most frequent for grades 3 to 6. These results are in line with the hypotheses that were based the RR theory (Karmiloff-Smith, 1990): the *growth* option refers to a less advanced level of RR, while the *different* option refers to a more advanced level. The *incomplete* and *mix* classes appeared in all grades. For the function model, however, the Wald criterion did not clearly demonstrate a relation between grade and class membership (*z* = −1.89, *p* = 0.06). The sum score of the 6 function items did relate to grade, *F*_(1, 314)_ = 29.26, *p* < 0.001, *R*^2^ = 0.09.

#### Mental models “on the Spot?”

To examine whether a preceding generative drawing task increased the coherence of the responses for the ES items, we compared the two experimental groups: children completed the drawing task followed by the questionnaire and children for whom this sequence was reversed.

Coherence was defined as the proportion of responses for ES items that were equivalent, that is, representing the same mental model. A *t*-test comparing the groups showed a small, but significant difference for coherence [*t*_(302)_ = 2.49, *p* = 0.01, Cohen's d = 0.28]. Children who completed the drawing task first had a higher coherence score (*M* = 0.84, *SD* = 0.17) than children who completed the questionnaire first (*M* = 0.79, *SD* = 0.19).

The same effect was present when analyzing whether the sequence of tasks affected the model children adhered to (Wald statistic in a logistic regression: *z* = 2.41, *p* = 0.02). If the drawing task preceded the questionnaire, children were assigned to a class with greater coherency: a growth model was more frequent (37 vs. 25%) and a mixed model was less frequent (16 vs. 24%).

#### Drawing task and comparison of methodologies

**Figure 4:**
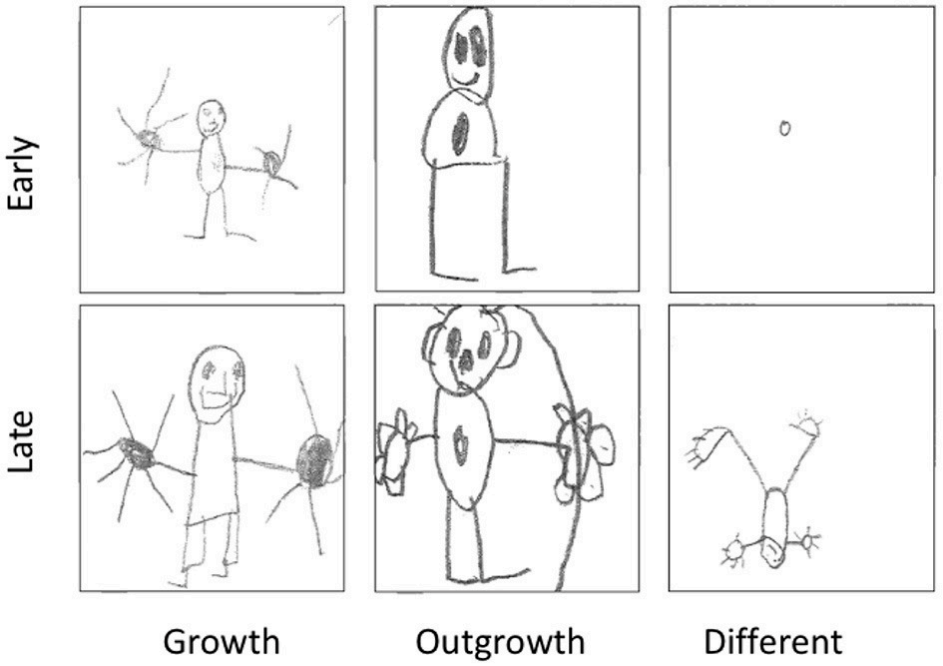
Examples of early and late stage drawings classified as *growth, outgrowth*, and *different*.

We classified the drawings into three categories (Figure 4 shows examples of drawings from three different children). The *no-change* and *growth* category were merged, because in the analysis of ES questionnaire items the four response options were analogously reduced to three response options. Moreover, scoring *growth* in the sometimes miniature drawings was difficult. Inter-rater reliability of classifications based on the three categories (*no change/ growth, outgrowth, different*) ranged from 92 to 96%. Cohen's Kappa ranged from 0.84 to 0.89.

There was no relation between grade and the drawings classification. To see whether agreement existed between the different tasks, we compared the drawings classification with the classification based on the questionnaire, but did not find a relation.

#### Conclusion

As latent class analysis of forced-choice questionnaire responses detected three subgroups of children showing coherent responses (either correct or incorrect), it was concluded that mental models about early fetal shape existed. The first model, the *growth* model, which was most frequently found in the early grades, assumes that the early fetus is smaller than a newborn baby. The second model, the *outgrowth* model, which was present in all grades in small percentages, assumes that some body parts are and some body parts are not present during early fetal development. That is, development consists of new body parts growing out of existing body parts, analogous to a growing plant with outgrowing branches. The third model, the *different* model, which was dominant in the highest grades, assumes that the early fetus looks very different from a newborn baby. A fourth group of children gave mixed, non-correlated responses. In contrast, the LCA of early and late-function items did not reveal multiple coherent naïve ideas other than the accurate idea. Most children responded that the functions in both early and late fetal development differ from post-natal functions. Only in concern to breathing we found multiple ideas. As the analysis did not detect multiple groups of children showing different, coherent responses on the function items, it was concluded that there is no evidence for mental models of bodily functions, other than the accurate idea that functions are different during fetal development. Moreover, the accuracy of responding increased somewhat with grade. This developmental pattern is consistent with the theoretical stance of fragmented knowledge.

The hypothesis that coherence of ideas increases by doing a generative task, such as drawing, was confirmed for the shape items. Straatemeier et al. (2008) did not find an effect of a generative task on the coherency of questionnaire responses. However, they did not find mental models as we do for the early shape items. Coherence of responses for the questionnaire was larger for the children who first drew two stages of fetal development. Eight percent more children yielded a mental model in that case, mostly a *growth* model. We did not find a clear correspondence between the models resulting from the different measurement methods. The results of the drawing task also did not relate to grade suggesting that this method was less useful in detecting mental models.

## Experiment 2

In Experiment 2 we compare the forced-choice questionnaire with structured interviews, which is another popular method in mental model studies. In contrast to forced-choice questions, open-ended interviews allow for generating detailed responses that were not anticipated during task design. The main research question is whether the mental models of early shape that we found with the questionnaire, can be replicated using the interview. Asking open-ended questions is argued to have a generative effect on mental models (Vosniadou et al., 2004). Hence, Experiment 2 also examines the effect of open-ended questions on the coherency of questionnaire responses.

### Method

#### Participants

Thirty-eight children were tested (age: *M* = 8.84, *SD* = 1.75) from a primary school providing regular education. The Review Board of the Faculty of the Social and Behavioral Sciences of the University of Amsterdam reviewed and approved all consent and study procedures. A written active consent procedure was used for all children because video recording were made during interviews. Parents received written letters with a reply sheet that could be given to the teacher in case the child was allowed to participate. The sample consisted of children between 6 and 12 years old: participants included 6 children from Grade 1, age 6–7 (*M* = 6.33, *SD* = 0.52); 6 children from Grade 2, age 7–8 (*M* = 7.33, *SD* = 0.60); 8 children from Grade 3, age 8–9 (*M* = 8.50, *SD* = 0.63); 8 children from Grade 4, age 9–10 (*M* = 9.38, *SD* = 0.67); 7 children from Grade 5, age 10–11 (*M* = 11.00, *SD* = 0.51); and 3 children from Grade 6, age 11–12 (*M* = 11.33, *SD* = 0.58).

#### Materials

The same materials were used as in Experiment 1: the forced-choice questionnaire and the drawing task on prenatal development. Additionally, an interview was conducted individually. The structure of the interview was similar to that of the questionnaire, with children being asked questions about the early and late stages of prenatal development. Six of the questions concerned shape (3 ES and 3 LS items) and four bodily functions (2 EF and 2 LF items) of the fetus. Additionally, two questions about the fetus as a whole were added, leading to a total of 12 questions. Questions were formulated to be open-ended. A maximum of two follow-up questions for anticipated responses was used to avoid repeated questioning and enhance rules of normal communication. To facilitate the child's understanding of the questions, a doll was used as a model for the newborn. An example of a shape item is: “*Look* [interviewer points at doll's hand], *this is what the hand of a baby looks like, when it has just been born. Does the baby have hands yet when the baby still has to stay in the belly of the mother for a long, long time?”* A confirming answer would lead to the question: “*What does the hand look like when the baby still has to stay in the belly of the mother for a long, long time?”* If the child answered that the fetus does not have hands yet, the interviewer would ask: “*So what does the baby have?”* The interview took about 10 min.

All interviews were video-recorded and scored afterwards. Questions were scored separately and independently. A list of responses was specified for each question, representing the core characteristics of the expected models. Children's shape responses were scored as (1) *no-change* response, (2) *growth* response, (3) *outgrowth* response, and (4) *different* response. Children's function responses were scored as: (1) *no-change* response, (2) *absence* response, and (3) *different* response.

#### Design and procedure

Children were tested with two sequences of the tasks. There was a fixed sequence for the tests administered in a classroom setting; all children first completed the drawing task and then the questionnaire. Children were either interviewed individually before (18 participants) or after the classroom tests (20 participants). The procedure of the classroom tests was the same as in Experiment 1.

### Results

#### General

The inter-rater reliability of the 10 interview items was 84% agreement on average (63 to 100%), which resulted in a Kappa of 0.73 (range 0.54 to 1.0). The internal consistency of the Shape and Function items in the interview with the response options taken as an ordinal variable (*no-change, growth, incomplete, different*) was 0.71, as expressed by Cronbach's Alpha. Non-parametric (Spearman's rho) correlations between items varied between 0.35 and 0.61 for the ES items, was 0.51 for the EF items, were between 0.44 and 0.53 for the LS items, and was 0.48 for the LF items. Table 3 shows the distributions of responses.

**Table 3:** Distributions of responses for the interview items in Experiment 2.

**Item**	**No-change**	**Growth**	**Outgrowth**	**Different**
**EARLY SHAPE**				
Hand	0	47.4	31.6	21.1
Leg	2.6	50.0	34.2	13.2
Ear	2.6	47.4	28.9	21.1
**EARLY FUNCTION**				
Drinking	19.4	0	5.6	75.0
Breathing	51.4	0	10.8	37.8
**LATE SHAPE**				
Hand	39.5	60.5	0	0
Leg	32.4	62.2	5.4	0
Ear	28.9	68.4	0	2.6
**LATE FUNCTION**				
Drinking	19.4	0	8.3	72.2
Breathing	44.4	2.8	13.9	38.9

*Frequencies are given as percentages of children (N = 38). From the 380 data points, 8 were missing*.

#### Mental models of bodily shape and function

As mentioned in the Method, children's responses to the interview questions were first scored according to the mental models that were expected beforehand, and were indeed detected in the questionnaire data, the *growth, outgrowth*, and *different* mental models. One of the interview questions was: “*Does the baby have hands yet when the baby still has to stay in the belly of the mother for a long, long time?*” An example of a *growth* response would be: “*Yes*, [interviewer asks what the hand looks like] *very small, smaller than this* [child points at the doll's hand], *a bit like this* [child marks an area as large as the doll's hand].” An example of an *outgrowth* response would be: “*No*, [interviewer asks what the baby does have] *it does have little arms, I think, from here* [child points to the wrist of the baby doll].” An example of a *different* response would be: “*I think so, but then a bit misshapen*, [interviewer asks the child whether she can explain what they would look like] *not very well, but I think a bit pudgy, just round, so to say, I think*.” (see Method for the exact phrasing of the question).

Due to the small number of participants it was not possible to apply LCA to the scored interview responses. To examine whether the interview revealed mental models, we analyzed the coherency of the scored responses. Hence, to examine coherency for the Shape items we counted the number of responses in agreement with each mental model (no-change/growth, outgrowth, different), and classified these as coherent if they all represented the same mental model. For the 3 ES items, 50% of the children showed coherent responses to these items in the interview, vs. 72% for the corresponding items in the questionnaire. For the four EF and LF items, for which we did not find mental models in the questionnaire, 44% of the children showed coherent responses in the interview, vs. 72% for the corresponding items in the questionnaire (all *different* responses). As the interview responses were not very coherent for both the Shape and Function items, the interview items were difficult to classify into mental models.

#### Comparing methodologies

To examine whether children adhered to the same mental model based on the interview and questionnaire data, we classified children's questionnaire responses. The questionnaire data of two participants was incomplete, and therefore the analyses including the questionnaire data were based on 36 participants. We based this classification on the early shape latent class model from Experiment 1, by means of the posterior probabilities of the responses given the four classes of this model^2^.

We used the Experiment 1 early shape latent class model to classify responses instead of pattern matching to include the possibility that a response pattern was assigned to the mixed class. Based on the three ES items of the interview children were also classified to one of the four classes.

Comparing the classifications of children based on the questionnaire and based on the interview, a dependency was found [χ^2^(2) = 20.68, bootstrapped *p* < 0.01; See Table 4). However, this dependency resulted from the large growth class only. For the other cells in the cross-table we did not see overlap.

**Table 4:** Cross-table of the classification of children's questionnaire and interview responses.

**Questionnaire**	**Interview**				
	**Growth**	**Outgrowth**	**Different**	**Mix**	***N***
Growth	14	2	1	5	22 (61%)
Outgrowth	0	0	0	0	0 (0%)
Different	0	4	1	0	5 (14%)
Mix	1	4	3	1	9 (25%)
*N*	15 (42%)	10 (28%)	5 (14%)	6 (17%)	36

As in experiment 1, children's drawings were classified in three categories: *no change growth, outgrowth, different*. The classification based on the drawings was not related to the questionnaire classification or to the interview classification.

#### Mental models “on the Spot?”

For each child, the coherence of the questionnaire responses was expressed as the proportion of early shape responses that were equivalent, that is, representing the same mental model. There was no effect of the sequence of tasks (interview before or after the drawing and questionnaire) on the coherence score, nor on the mental models (based on the questionnaire) to which the children adhered.

#### Conclusion

Interview responses were less coherent than questionnaire responses. The interview responses did not support the existence of the expected mental models. A significant dependency was found between the classification based on the interview and the classification based on the questionnaire. The correspondence, however, was only due to the large *growth* class.

Vosniadou et al. (2004) stated that asking open-ended questions would have a generative effect on the construction of mental models, just as drawings do. In Experiment 2 the drawings always preceded the questionnaire, which was shown to have a generative effect on the questionnaire in Experiment 1. Coherency of the ES items of the questionnaire in Experiment 2 (*M* = 0.83, *SD* = 0.19) was indeed comparable to the coherency of these items in Experiment 1 when preceded by the drawings (*M* = 0.84, *SD* = 0.17). Administering the interview did not have an additional generative effect on the coherence of children's questionnaire responses for the ES items.

## Discussion

### Mental models of prenatal development

One of the main questions in the literature about children's naïve ideas about the world is whether children form (incorrect and correct) mental models or whether their naïve ideas consist of incoherent, fragmented knowledge. We studied this issue for the case of mental models of prenatal development. Applying latent class analysis to forced-choice questionnaire data allowed for testing the existence of coherent response patterns over multiple items, which is important evidence for the existence of mental models (Straatemeier et al., 2008). Experiment 1 showed that for most children (78%) their naïve ideas about changes in the shape of a fetus' body were coherent. We detected two groups of children with coherent patterns of incorrect responses, that is, incorrect mental models. These children believed that the body only *grows* between early and late stage of prenatal development (30%), that most body parts *grow out* of the body (6%). Note that the outgrowth model was not found in Experiment 2, but given that this group was small in Experiment 1, this is not completely unexpected. In addition, we detected a group of children with a coherent pattern of correct responses. These children believed that the shape of the body is *different* between early and late stage of prenatal development (42%), which is an indication for having a (at least partially or superficially) correct mental model. Although the applied statistical approach does not distinguish between a coherent mental model and correct knowledge for separate facts, we argue that in this specific domain, fetal development, such a response pattern is more likely an indicator of the existence of a mental model. In their daily lives, children may on occasion see an image of a fetus in a book, on television, or on an ultrasound, but for most children these could be considered rare occasions. Therefore, the performance of the relatively large group of children (42%) with the coherent pattern of correct responses could not be explained by the children having factual knowledge of multiple body parts (fetal hand, hand, leg, ear, foot, arm, eye) and being able to demonstrate this knowledge on items they had never seen before. Last, 22% of the children showed an unsystematic mix of two types of responses: body parts grow or undergo important changes. There are various possible reasons for this incoherence: some pictures of the test seemed implausible to children; children's ideas were instable; or children guessed between two most plausible response options. Based on both the detection of two incorrect mental models, as well as the detection of a group with a coherent correct response pattern that could best be explained by a (at least partially or superficially) correct mental model, we conclude that the results of this study demonstrate the existence of mental models of prenatal body shape. This conclusion is consistent with the results of Rosengren et al. (1991) about children's acceptance of natural transformations. According to Rosengren et al. young children know that animals get larger with age, but only older children and adults accept rather dramatic changes in shape. Grade-related individual differences in mental models could be due to different observations that children make during childhood. Different types of shape changes are more or less familiar in the natural environment. That is, different mental models resemble natural growth processes that children could have observed in themselves (*growth*), plants (*outgrowth*), or for example butterflies (*different*). Menendez et al. (2017) show that children can generalize observations of change into a different shape to other animals (from a ladybug to other insects). French et al. (2018) argue that cognitive constraints play a role in the types of changes that children accept to occur during the developmental processes.

For bodily functions the results were different. Most children believed that fetal bodily functions were different from the functions of a newborn (66%). For breathing only, some children (23%) believed that this function is the same in the fetus and the newborn, and some children (11%) believed that the fetus does not need something from the air at all. Hence, we did not find evidence for incorrect mental models of fetal bodily functions. We only detected one coherent response pattern concerning the changes of bodily functions during prenatal development, consistent with the correct *different* model. Although it is correct that bodily functions are different in a fetus compared to a newborn, the *different* responses do not necessarily indicate that most children had a scientifically acceptable mental model. In the questionnaire we did not test children's ideas about bodily functions in more detail because a coherent detailed idea describing change in multiple functions simultaneously is not very likely (or thinkable). Hence, we conclude that we found no evidence for children to have incorrect mental models on the level of change of bodily functions in general (drinking, breathing, keeping warm). Whereas, children could reason about prenatal changes of shape analogously to growth processes observed with other living things, this is more difficult for bodily functions. Drinking, for example, is a very stable function that only changes in early child development from drinking from a bottle or breasts to a cup, but also adults still drink. It is not clear how to use these observations to predict “*how a fetus gets drink*.” Menendez et al. (2017) argue that a *growth* model of development is an indication of a featural stability bias as a component of the essentialist reasoning. A featural stability bias is a systematic tendency to assume that features of living creatures are stable over time. Reasoning about the perceptual features of familiar creatures, this bias was observed mostly in young children (Menendez et al., 2017). The current study confirms a featural stability bias in young children's reasoning about bodily shape (the *growth* model). But a featural stability bias (a *growth* model) was not observed (systematically) when children reasoned about bodily functions.

The conclusions we drew about evidence for the existence of children's mental models of fetal development were predominantly based on the questionnaire data. We did so for several reasons. Firstly, we drafted hypotheses specifying specific response patterns based on representational redescription theory (Karmiloff-Smith, 1990) and previous research (e.g., Zoldosova and Prokop, 2007), and with the questionnaire we detected these specific response patterns (mental models), while it would have been very unlikely to detect these on the basis of chance. In contrast, with the interview we detected these response patterns to a lesser extent. Secondly, in line with the first argument, our hypotheses indicated the existence of coherent response patterns, and the coherence of the responses in the interviews was considerably less than for the questionnaire. Thirdly, the questionnaire had a higher internal consistency than the interview. Fourthly, several observations suggested that the interview possibly did not measure children's mental models of fetal development, but their abilities to construct narratives from fragmented knowledge (diSessa, 2008). The finding that children almost never responded with “I don't know” (a maximum of 2% of the responses were omitted), which is remarkable given that young children appear to know very little details about human insides (Morris et al., 2000), supports this explanation. Further research into this explanation is required as the use of interviews and drawings as formative assessment methodologies might be interesting from an educational perspective. In Experiment 1 we see that children's responses on the questionnaire were more coherent after drawing different phases of prenatal development. Hence, these methods seem to stimulate children's thinking about prenatal development, creating good opportunities for teachers to present new knowledge about the topic (Schwartz and Sadler, 2007).

### Representational flexibility

The mental models that we found for early shape development are consistent with Karmiloff-Smith's (1992) ideas about representational flexibility. Representational flexibility is assumed to occur within specific topics (i.e., micro-domains) throughout development, but the overall sequence of introduced modifications is believed to be domain general. Prenatal development is an interesting topic for the study of representational flexibility, as children need to imagine how a baby would look like in an earlier stage of development. That is, they have to modify their concept of a baby such that it is consistent with what they know about prenatal development and a newborn baby. The question is how flexible children are in adapting their concept. Indeed, the grade-related results on the questionnaires are in agreement with the specific sequence of phases in representational flexibility. Karmiloff-Smith's original ideas about representational redescription were based on drawings of human beings. Remarkably, our results based on children's drawings did not reveal a significant relation between representational flexibility and grade.

Whether representational flexibility is dependent on procedural constraints or is more dependent on conceptual rigidity is a debated issue in literature with both positive (Picard and Vinter, 2007) and negative evidence (Spensley and Taylor, 1999; Barlow et al., 2003; Hollis and Low, 2005). According to Karmiloff-Smith (1990) the procedural constraint in the first phase of representational redescription is that young children's internal representations are dependent on a sequentially fixed list. Representational flexibility increases in subsequent phases because the constraints are relaxed. Results of the study by Picard and Vinter (2007) are in line with Karmiloff-Smith's theory. They find that rigidity in routine development constitutes a sequential constraint that limits inter-representational change. When this sequential constraint is relaxed, it is likely to be one of the factors leading to inter-representational flexibility. In the present study we found evidence that the representational redescription was more dependent on conceptual rigidity. Hence, it was not only dependent on procedural constraints, i.e., drawing skills. An interesting next step would be to test children's conceptual rigidity in multiple domains simultaneously to test underlying factors of this rigidity, such as whether the concept was part of long-term memory or only recently created (see also Karmiloff-Smith, 1999).

### Methodological issues

The reason for the use of a forced-choice questionnaire was that this methodology makes it possible to measure coherence in responses, which we took as a defining criterion for establishing evidence for mental models. A point of criticism of this methodology could be that due to the limited response options children are more likely to respond according to a specific model, increasing the chance of the researcher finding a coherent model. However, it appears that the use of forced-choice questionnaires in investigating children's mental models does not necessarily lead to finding coherent models (Straatemeier et al., 2008). Moreover, unlike the shape related items, responses on the function items did not reveal multiple coherent models. A second point of criticism could be that the questionnaire was composed of adapted photographs. This could have led to a response bias; children might have just picked the most unusual picture. The lack of variation in the responses to the late-shape items provides evidence against the existence of such a bias. Here, children chose the *no-change* and *growth* response for all items, which photographs look like the newborn.

The use of LCA made it possible to detect a subgroup of children with mixed, incoherent responses. For the early shape items only a few children showed a more or less coherent pattern of outgrowth responses. Nevertheless, identifying this subclass of children contributes to the best describing, most parsimonious model. Hence, the methods that we applied to detect mental models allowed for establishing evidence for common mental models, rare mental models, and the absence of a mental model within the same group of children.

### Conclusion

Taking into account the scarcity of previous experimental work on mental models of prenatal development, our study adds substantial insights on the topic. There is a variety of grade related mental models of prenatal shape development that exist during childhood. There was no evidence for such a variety of mental models about bodily functions describing change in multiple functions simultaneously. These findings imply that, dependent on the specific topic, children reason from separate facts or from general principles. In the area of fetal development, children reason from separate facts when the topic concerns bodily functions (cf. Zoldosova and Prokop, 2007), but from general principles when the topic concerns bodily shape, i.e., transformations that are acceptable for shapes or animals (Rosengren et al., 1991). A suggestion for future research would therefore be to relate children's knowledge of fetal development to their knowledge of bodily development or, more general, biological growth. In addition, more systematic studies on the relation between available sources of knowledge and the type of knowledge that children construct from these sources could provide more general insight in the development of knowledge.

## Ethics statement

This study was carried out in accordance with the recommendations of the national code of ethics for research in the social and behavioral sciences, Faculty Ethics Review Board (FMG-UvA). The protocol was approved by the Faculty Ethics Review Board (FMG-UvA). In accordance with the Declaration of Helsinki, legal authorities (mostly parents) of all subjects who were video recorded gave written informed consent; for all other subjects we followed an opt-out consent procedure for their legal authorities (mostly parents).

## Author contributions

TvS, SvE, RF, BvB, and MR conceived the ideas, designed methodology, and prepared test materials; SvE collected the data; TvS and MR analyzed the data; TvS and MR led the writing of the manuscript. All authors contributed critically to the drafts.

### Conflict of interest statement

The authors declare that the research was conducted in the absence of any commercial or financial relationships that could be construed as a potential conflict of interest.

## Footnotes

^1^The workshop was LOOL, which stands for “leren onderzoekend en ontwerpend leren,” Dutch for “enquiry based learning and learning by design.” The project was led by Marja van Graft and executed by Rooske Franse in 2005 and 2006.

^2^The classification of individuals depends both on conditional and unconditional parameters of the model. We assume that the estimates of conditional parameters are the same for Experiment 1 and Experiment 2 children. However, the unconditional parameters depend on the distribution of age and/or grades within the group, which we do not assume to be equal across experiments. Therefore, we estimated the latent class model again with grade added as a covariate on the posterior probabilities. Grade was significantly related to the model classification of the questionnaire [likelihood difference between models is: χ^2^(2) = 7.07, *p* < 0.03]. Almost the same classification of Experiment 2 children was found on basis of this model. Only one subject was classified as having a *growth* model instead of *mixed* ideas. For reasons of simplicity, we worked with the simple model that was explained in the text.
