# Supplementary material for: Children's Mental Models of Prenatal Development
Source: Front Psychol. 2018 Oct 1;9:1835. doi: 10.3389/fpsyg.2018.01835 (PMC6174239; doi:10.3389/fpsyg.2018.01835)
Supplement: Supplementary file 1 [file Data_Sheet_1.PDF]

Supplementary materials

Test materials (English and Dutch) as used (in Dutch) in the following article

Children's Mental Models of Prenatal Development

Tessa J. P. van Schijndel[1], Sara E. van Es[2], Rooske K. Franse[2,3], Bianca M.C.W. van Bers[2], Franse[2,3], Maartje E. J. Raijmakers[2,4*]

[1] Research Institute of Child Development and Education, University of Amsterdam, Amsterdam, The Netherlands

[2] Developmental Psychology, Dept. of Psychology, University of Amsterdam, Amsterdam, The Netherlands.

[3] NEMO science museum, Amsterdam, The Netherlands.

[4] Educational Studies, Faculty of Behavioral and Movement Sciences, Free University Amsterdam, Amsterdam, The Netherlands.

*** Correspondence:**
Maartje Raijmakers
m.e.j.raijmakers@uva.nl

Protocol for classroom-presented questionnaire prenatal development
Now we are going to start with the questionnaire.

I am going to ask you some questions. The questions are presented on screen. They are also in the booklet that lies in front of you. Circle the answer option you think is correct.

First, write on paper your name, age, and class.

Now we are going to start. You are only allowed to turn the page when I tell you. I first show you the question on screen, only then are you allowed to turn the page. Wait with turning the page until I give you permission.

Practice question
(PPNT-vb-question) The first question is a practice question.

The question is: *which pictures is most similar to a cat?*
Now turn the page.
Circle the correct picture.

Does everyone understand how you are supposed to answer a question? (answer questions if necessary)

Now I will ask you questions about babies that still are in the belly of their mother. First, I will ask you a couple of questions about a baby that is in the belly of its mother, when the baby has to stay in the belly for a very long time.

Now we are going to start with the questions. Wait with turning the page until I give permission.

(PPNT-HAND)
1) This is how a **hand** of a newborn baby looks like. What does the hand of a baby look like when the baby has to stay in the belly of the mother for a very long time?
a) A hand that looks exactly the same (cg-no growth)
b) A hand that looks exactly the same but it is smaller (cg-with growth)
c) A hand that looks like the hand of a newborn baby but does not already have all five fingers (ci)
d) A hand that does not look like the hand of a newborn baby. It has a different shape. (cv-v)

(PPNT-LEG)
2) This is how a **leg** of a newborn baby looks like. What does the leg of a baby look like when the baby has to stay in the belly of the mother for a very long time?
a) A leg that does not look like the hand of a newborn baby. It has a different shape (cv-v)
b) A leg that looks exactly the same (cg-no growth)
c) A leg that looks exactly the same but is smaller (cg-with growth)
d) A leg that looks like the leg of a newborn baby but as yet only the upper leg is present (ci)

(PPNT-EAR)
3) This is how an **ear** of a newborn baby looks like. What does the ear of a baby look like when the baby has to stay in the belly of the mother for a very long time?

a) There is no ear (ci)
b) An ear that looks exactly the same but is smaller (cg-with growth)
c) An ear that does not look like the ear of a newborn baby. The ear has a different shape. (cv-v)
d) An ear that looks exactly the same (cg-no growth)

(PPNT-FOOT)
4) This is how a foot of a newborn baby looks like. What does the foot of a baby look like when the baby has to stay in the belly of the mother for a very long time?

a) A foot that looks exactly the same (cg-no growth)
b) A foot that does not look like the ear of a newborn baby. The foot has a different shape. (cv-v)
c) A foot that looks exactly the same but is smaller (cg-with growth)
d) A foot that looks like the foot of a newborn baby but does not already have all five toes (ci)

(PPNT-ARM)
5) This is how an **arm** of a newborn baby looks like. What does the arm of a baby look like when the baby has to stay in the belly of the mother for a very long time?

a) An arm that looks like the arm of a newborn baby but as yet only the upper arm is present (ci)
b) An arm that looks exactly the same but is smaller (cg-with growth)
c) An arm that looks exactly the same (cg-no growth)
d) An arm that does not look like the ear of a newborn baby. The arm has a different shape. (cv-v)

(PPNT-EYE)
6) This is how an **eye** of a newborn baby looks like. What does the eye of a baby look like when the baby has to stay in the belly of the mother for a very long time?

a) An eye that does not look like the eye of a newborn baby. The eye has a different shape. (cv-v)
b) An eye that looks exactly the same (cg-no growth)
c) An eye that looks exactly the same but is smaller (cg-with growth)
d) There is no eye (ci)

(PPNT-DRINK)
7) When a baby is born it gets milk from the mother through its mouth. This is how a baby **drinks**. How does a baby drink when the baby has to stay in the belly of the mother for a very long time?

a) The baby in the belly drinks through its mouth, just like a newborn baby (cg)
b) The baby in the belly does not need to drink (ci)
c) The baby in the belly drinks through a tube that is attached to the belly button (cv-f)

(PPNT-WARM)
8) When a baby is born it needs clothes to **stay warm**. How does a baby stay warm when the baby has to stay in the belly of the mother for a very long time?

a) The baby in the belly does not need to stay warm (ci)
b) The baby in the belly stays warm because the belly of the mother keeps it warm (cv-f)
c) The baby in the belly wears clothes, just like a newborn baby (cg)

(PPNT-AIR)
9) When a baby is born it needs to **breath air** to stay alive. How does a baby breath when the baby has to stay in the belly of the mother for a very long time?
a) The baby in the belly gets air through the blood of the mother (cv-f)
b) The baby in the belly breaths air, just like a newborn baby (cg)
c) The baby in the belly does not need air (ci)

Last phase:
Now I will ask you a couple of questions about the baby in the belly of its mother, just before the baby is born. The baby has been in the belly for quite some time and will almost be born.

(PPNT-HAND)
10) This is how a **hand** of a newborn baby looks like. What does the hand of a baby look like just before the baby is born?
a) A hand that does not look like the hand of a newborn baby. It has a different shape. (cv-v)
b) A hand that looks exactly the same (cg-no growth)
c) A hand that looks exactly the same but is smaller (cg-with growth)
d) A hand that looks like the hand of a newborn baby but does not have all five fingers (ci)

(PPNT-LEG)
11) This is how a **leg** of a newborn baby looks like. What does the leg of a baby look like just before the baby is born?
a) A leg that looks like the leg of a newborn baby but only the upper leg is present (ci)
b) A leg that does not look like the hand of a newborn baby. It has a different shape (cv-v)
c) A leg that looks exactly the same (cg-no growth)
d) A leg that looks exactly the same but is smaller (cg-with growth)

(PPNT-EAR)
12) This is how an **ear** of a newborn baby looks like. What does the ear of a baby look like just before the baby is born?
a) An ear that looks exactly the same (cg-no growth)
b) There is no ear (ci)
c) An ear that looks exactly the same but is smaller (cg-with growth)
d) An ear that does not look like the ear of a newborn baby. The ear has a different shape. (cv-v)

(PPNT-DRINK)
13) When a baby is born it gets milk from the mother through its mouth. This is how a baby **drinks**. How does a baby drink just before the baby is born?

a) The baby in the belly drinks through a tube that is attached to the belly button (cv-f)
b) The baby in the belly drinks through its mouth, just like a newborn baby (cg)
a) The baby in the belly does not need to drink (ci)

(PPNT-WARM)
14) When a baby is born it needs clothes to **stay warm**. How does a baby stay warm just before it is born?
a) The baby in the belly wears clothes, just like a newborn baby (cg)
b) The baby in the belly does not need to stay warm (ci)
c) The baby in the belly stays warm because the belly of the mother keeps it warm (cv-f)

(PPNT-AIR)
15) When a baby is born it needs to **breath air** to stay alive. How does a baby breath when the baby has to stay in the belly of the mother for a very long time?
a) The baby in the belly does not need air (ci)
b) The baby in the belly gets air through the blood of the mother (cv-f)
c) The baby in the belly breaths air, just like a newborn baby (cg)

That was the last question.

Questions

Name: ____

Age: ____

Year: ____

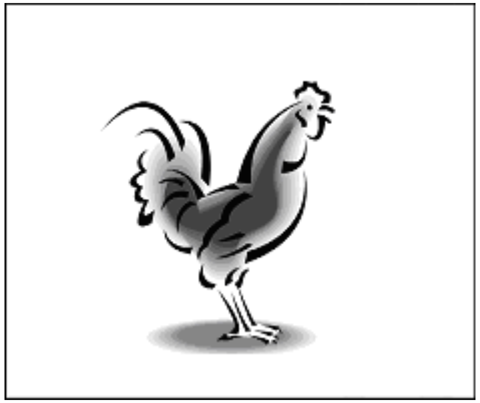
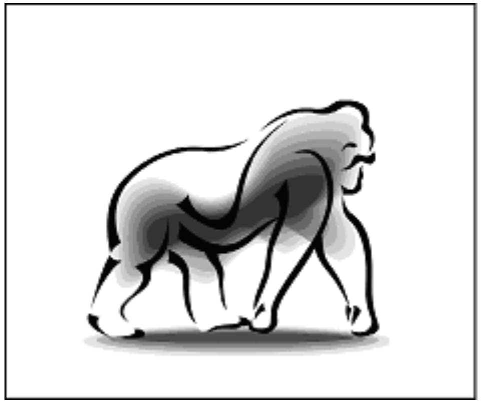
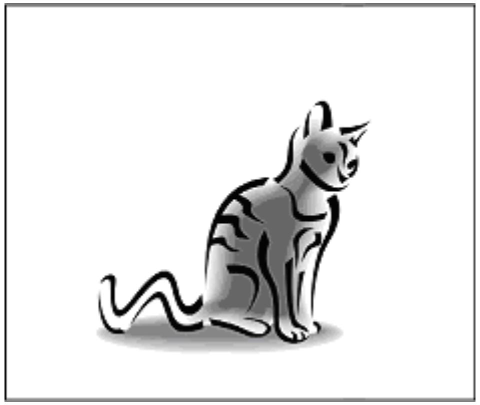

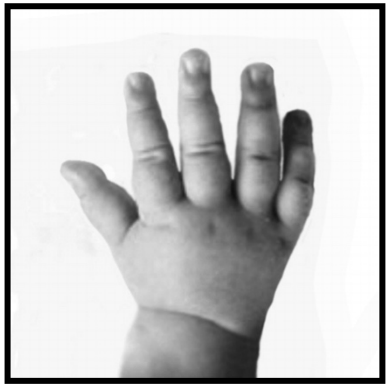

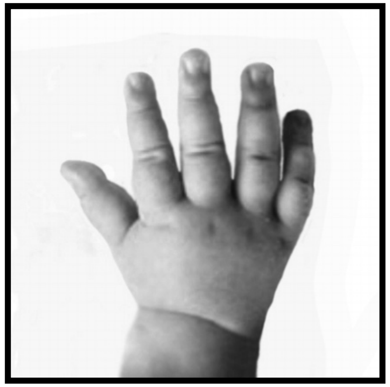

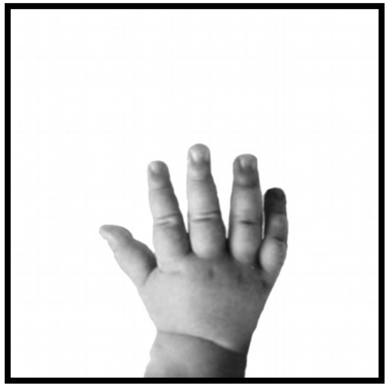

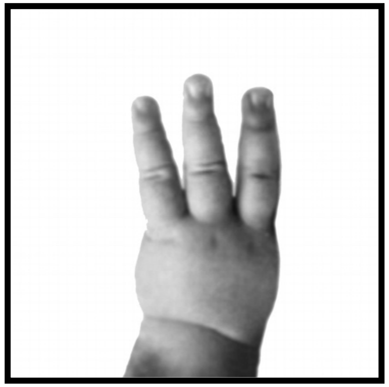

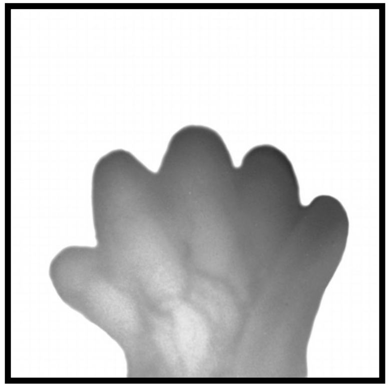

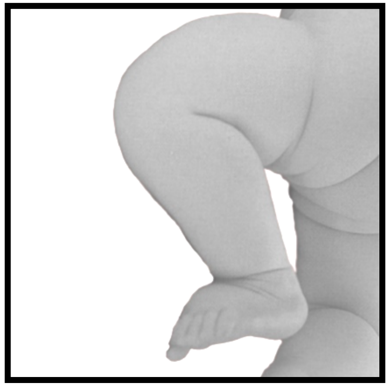

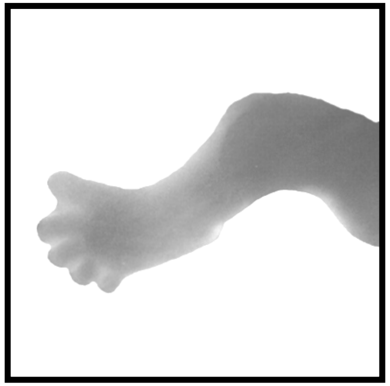

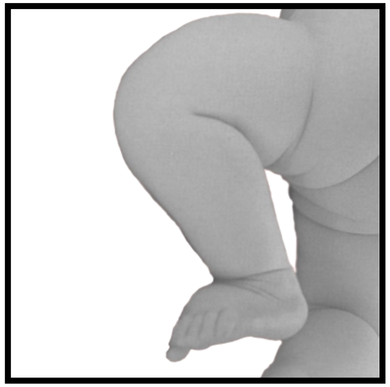

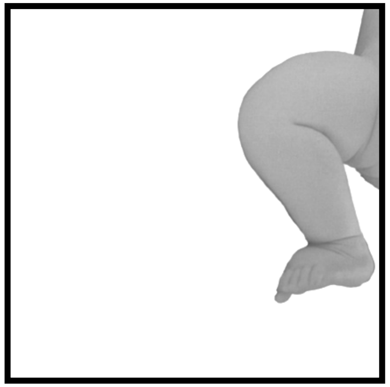

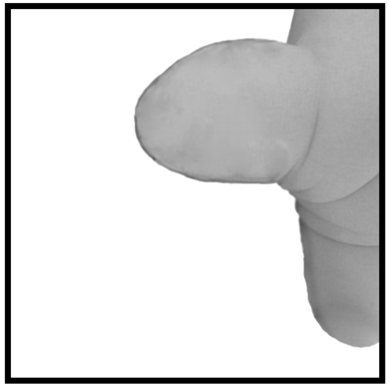

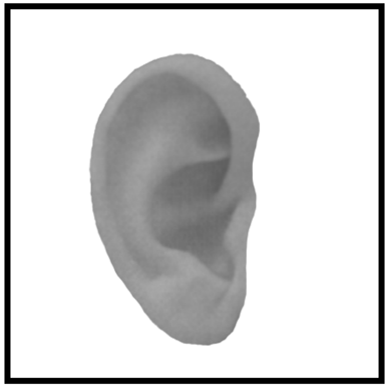

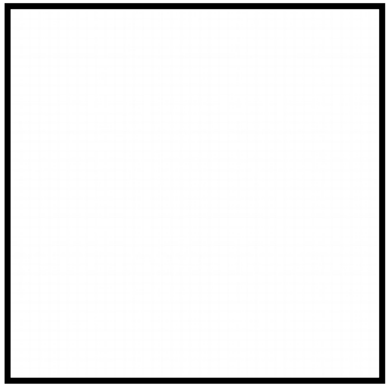
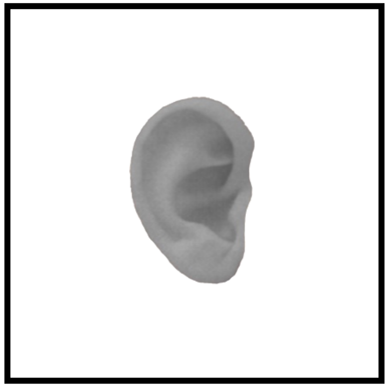
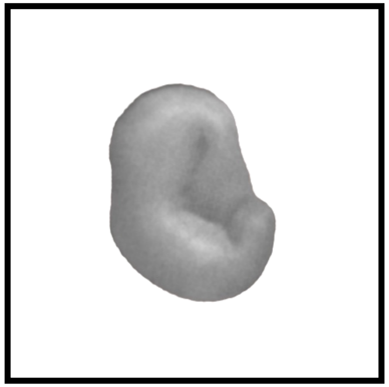
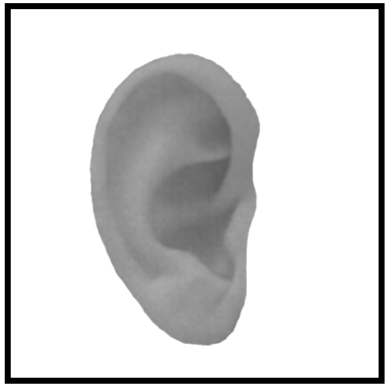

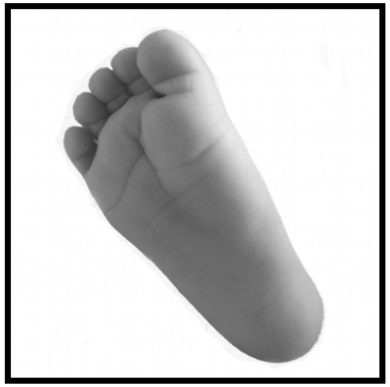

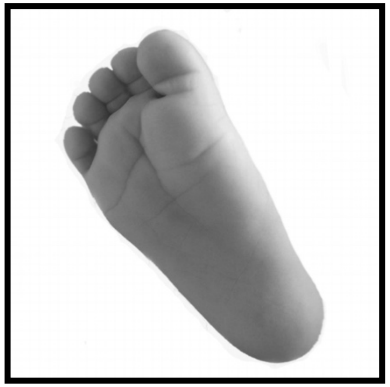

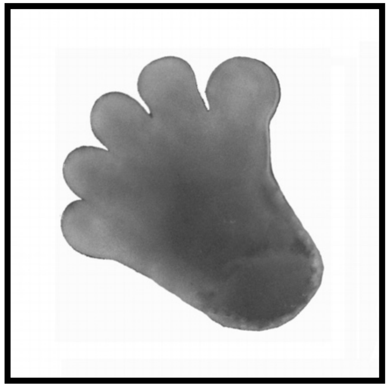

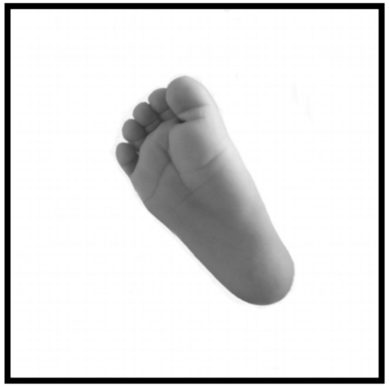

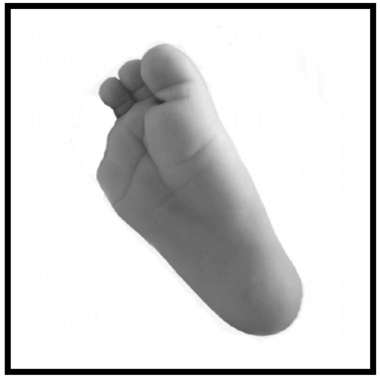

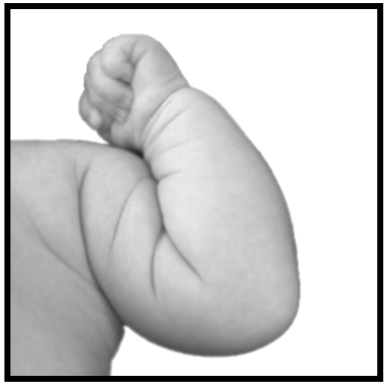

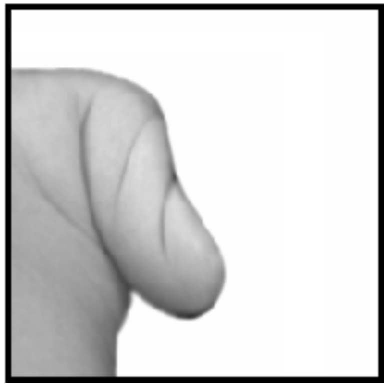

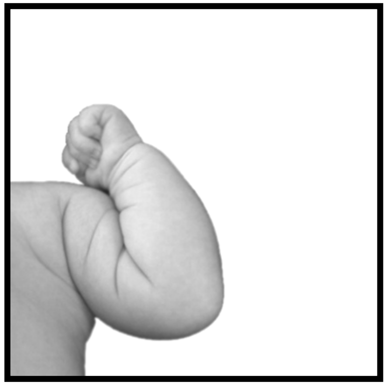

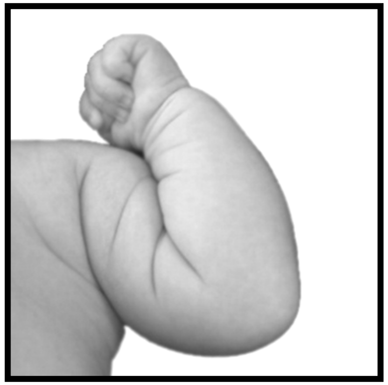

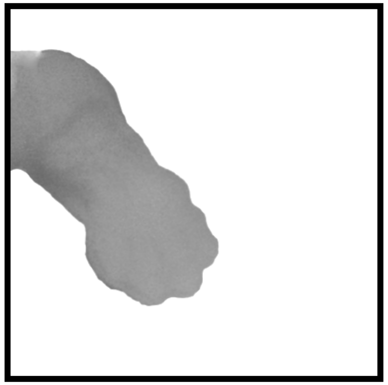

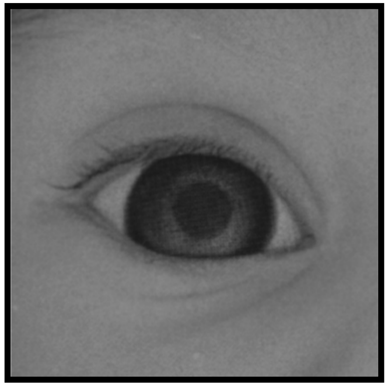

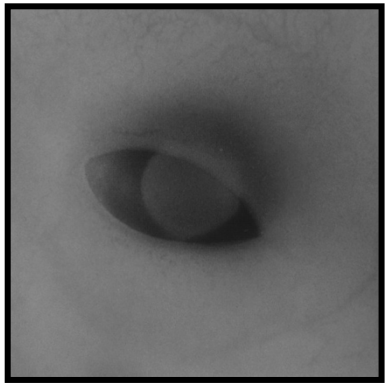

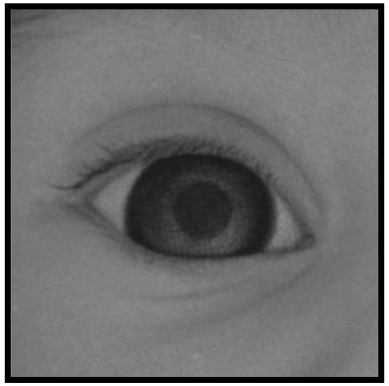

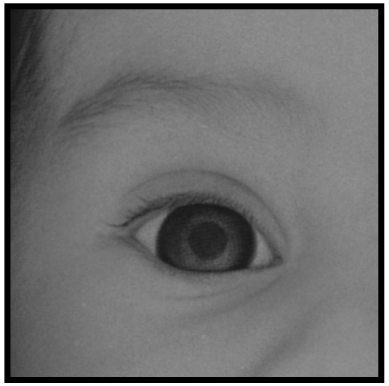

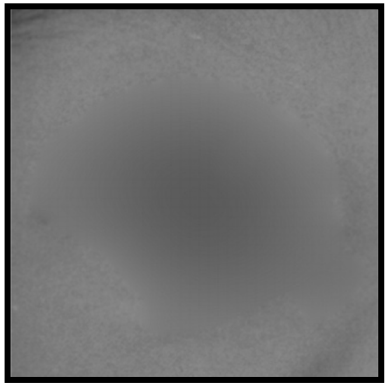

Drinking?
Milk through mouth

Milk through mouth

No drinking

Tube

Stay warm?

Clothes

No need to stay warm

Mother belly

Clothes

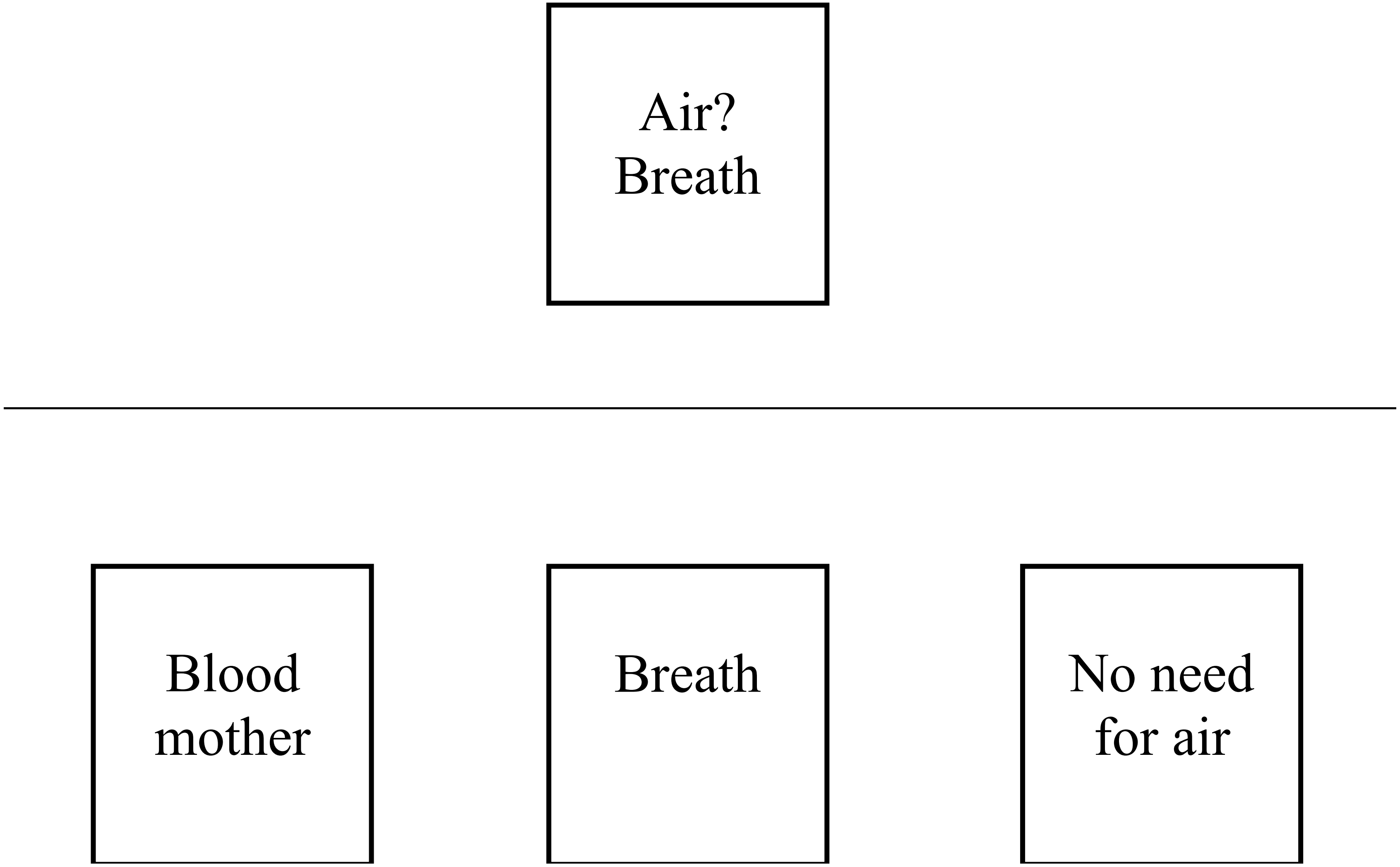
Air?
Breath
Blood
mother
Breath
No need
for air

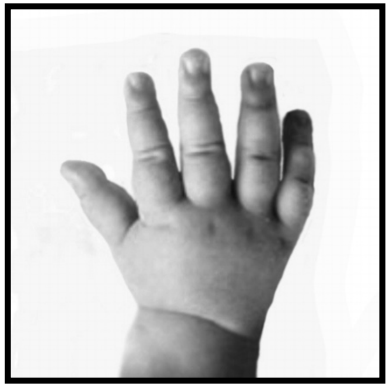

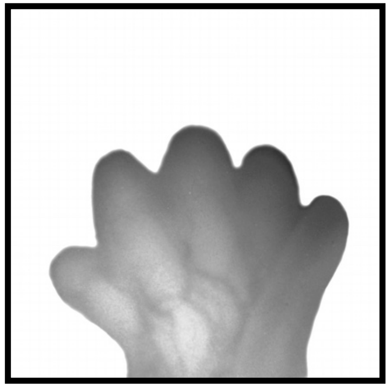

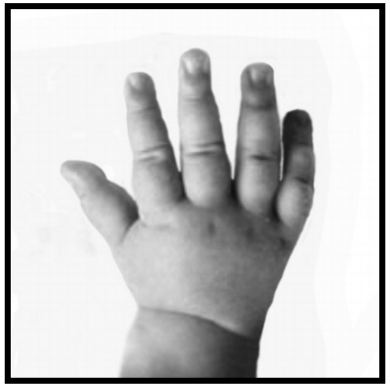

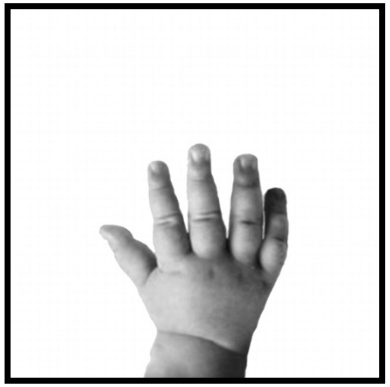

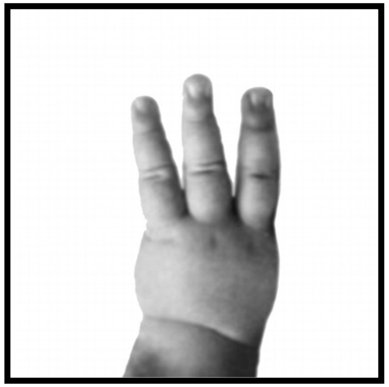

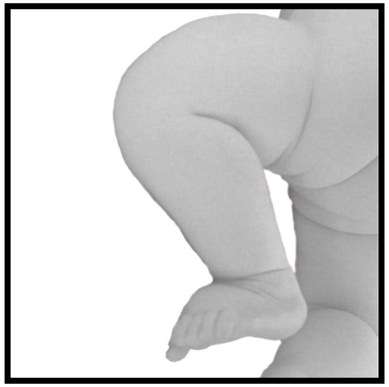

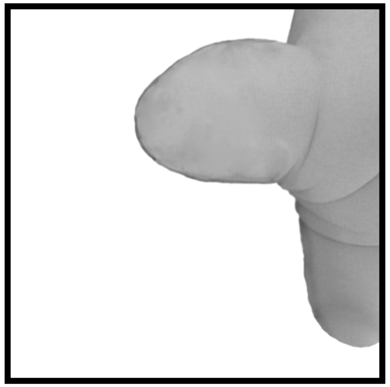

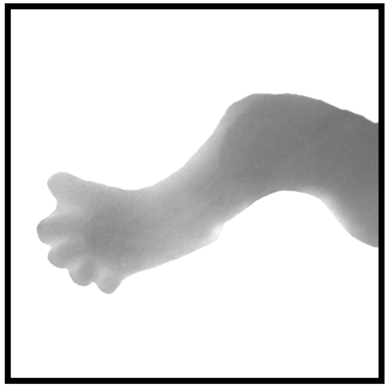

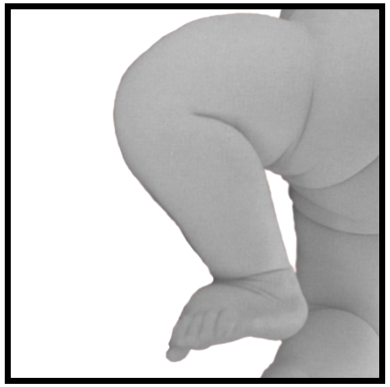

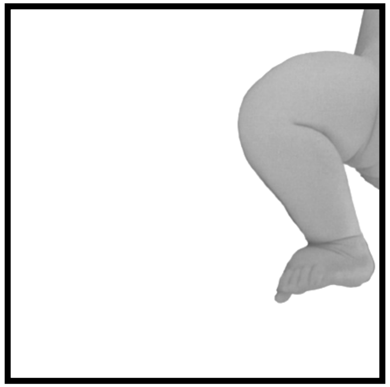

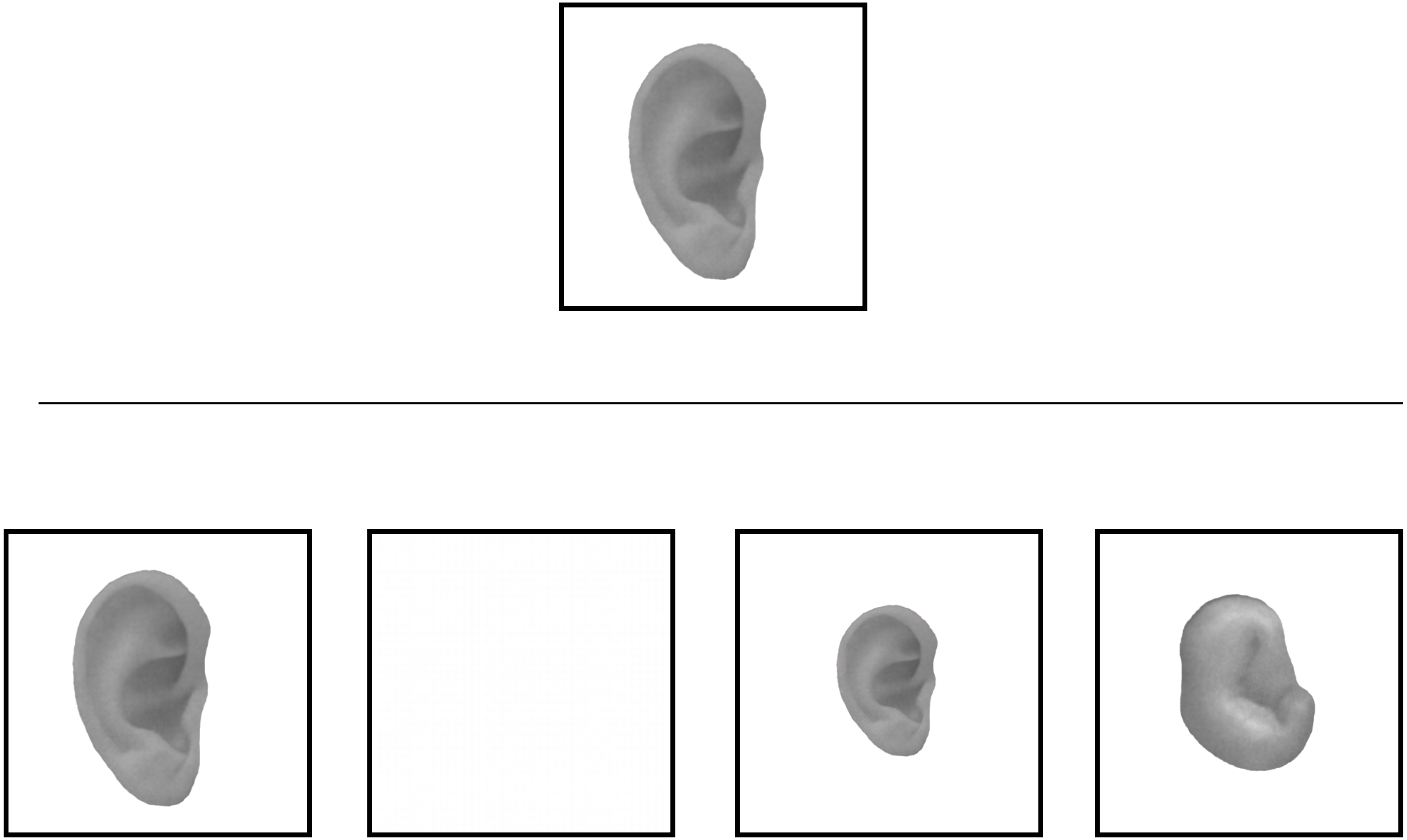

Drinking?
Milk through mouth

Tube

Milk through mouth

No drinking

Stay warm?

Clothes

No need to stay warm

Mother belly

Clothes

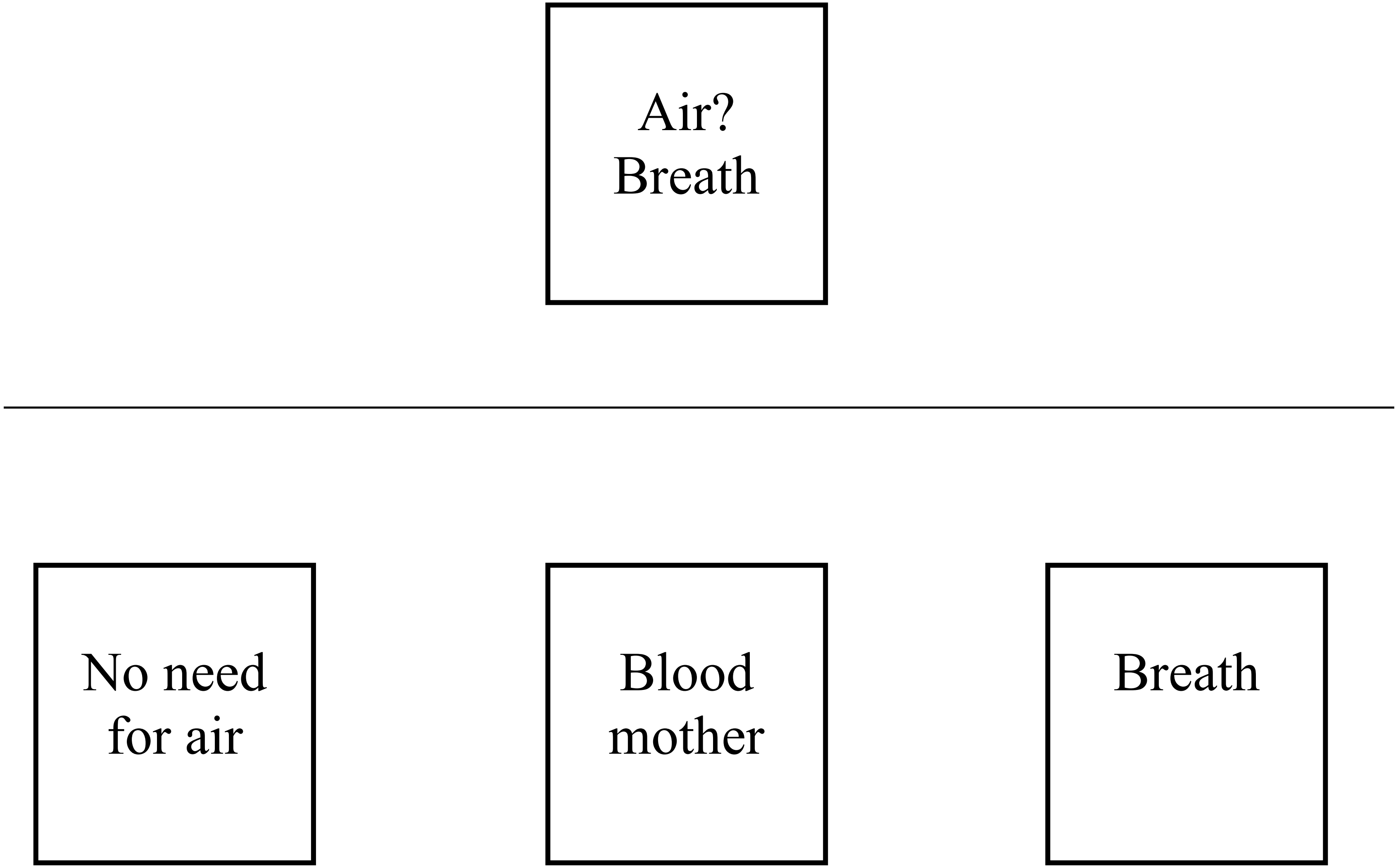
Air?
Breath
No need
for air
Blood
mother
Breath

Instructie bij het onderzoek
Tekening-VL-Nepmens

Hallo allemaal. Ik ben Sara van Es en ik ben een onderzoeker van de Universiteit van Amsterdam. Ik ben hier omdat wij het leuk vinden om er achter te komen hoe kinderen over sommige dingen denken. Dit keer gaat dat over baby's. Zijn er hier kinderen in de klas die een broertje of een zusje hebben? Dan weten jullie vast wel dat een baby, voordat deze geboren wordt, een hele lange tijd in de buik van de moeder zit.

Ik ga jullie vragen om drie dingen te doen. Ik ga jullie vragen om eerst een tekening te maken. Dan om wat vragen te beantwoorden. Als laatst mogen jullie weer een tekening maken. Je krijgt nergers een cijfer voor. Je mag niet bij elkaar kijken.

(1) Baby-tekening
We beginnen met de tekening. Jullie hebben allemaal een blaadje gekregen, zoals deze (laten zien op beamer). Zet op het blaadje je voornaam en je achternaam. Wacht met tekenen totdat ik zeg dat je mag beginnen.

Op het blaadje staan twee plaatjes van moeder met een leeg vierkantje op haar buik. In de lege vierkantjes mogen jullie zo meteen een tekening maken. In het derde vlak staat de moeder met een pasgeboren baby. Maar voordat een baby geboren wordt moet hij heel lang in de buik van de moeder zitten. Jullie mogen zo meteen 2 babies tekenen. 1 baby die nog heel lang in de buik moet zitten en 1 baby die er al bijna uit komt.

We gaan beginnen met de eerste tekening. Teken in het eerste lege vierkantje een baby in de buik van de moeder, **als de baby nog een hele lange tijd in de buik van de moeder moet zitten**. Teken hoe de baby eruit ziet, alsof je in de buik kan kijken. Jullie krijgen hiervoor tien minuten. Je mag nu beginnen. (tijd bijhouden! 10 minuten)

Na 10 minuten: We gaan nu beginnen met de tweede tekening. Teken in het tweede lege vierkantje een baby in de buik van de moeder, **vlak voordat de baby geboren wordt**. Dan zit de baby dus al best lang in de buik van de moeder en wordt bijna geboren. Teken hoe het kindje eruit ziet, alsof je in de buik kan kijken. Jullie krijgen hiervoor tien minuten. Je mag nu beginnen. (tijd bijhouden! 10 minuten)

Na 10 minuten: Jullie mogen nu stoppen met tekenen. Ik kom de tekeningen ophalen en de vragenlijst uitdelen.

(2) Vragenlijst
Dan gaan we nu beginnen met de vragenlijst.

Ik zal jullie nu wat vragen stellen. De vragen staan op het grote scherm. Ze staan ook allemaal in het boekje dat jullie voor je hebben liggen. Zet telkens een cirkel om het antwoord dat jij denkt dat goed is.

Zet nu op het voorste blad je naam, je leeftijd en je klas.

Dan gaan we nu beginnen. Je mag telkens pas de bladzijde omslaan als ik zeg dat het mag. Ik laat eerst de vraag op het bord zien, daarna mag je je bladzijde omslaan. Wacht dus met het omslaan van de bladzijde totdat ik het zeg.

Voorbeeld vraag
(PPNT-vb-vraag) De eerste vraag is een oefenvraag. Hiermee kun je oefenen met het invullen van je antwoord.

De vraag is: *welk plaatje lijkt het meest op een kat?*
Sla nu de bladzijde maar om.
Zet nu een cirkel om het juiste plaatje.

Snapt iedereen hoe je je antwoord moet invullen? (eventuele vragen beantwoorden)

Nu ga ik jullie vragen stellen over baby's gaan die nog in de buik van de moeder zitten. Eerst stel ik jullie een aantal vragen over de baby in de buik van de moeder, als de baby nog heel lang in de buik van de moeder moet zitten.

Dan gaan we nu beginnen met de vragen. Wacht telkens met het omslaan van de bladzijde tot ik zeg dat het mag.

(Telkens EERST OP BEAMER, DAN PAS BLZ OMSLAAN!!)

(PPNT-HAND)
1) Zo ziet de **hand** van een baby'tje eruit als het net geboren is. Waar lijkt het handje van een baby het meest op als de baby nog een hele lange tijd in de buik van de moeder moet zitten?

a) Een handje dat er precies hetzelfde uitziet (cg-zonder groei)
b) Een handje dat er precies hetzelfde uitziet maar dat wel kleiner is (cg-met groei)
c) Een handje dat lijkt op het handje van een geboren baby, maar het heeft nog niet alle vingers (ci)
d) Een handje dat niet lijkt op het handje van een pasgeboren baby. Het heeft een andere vorm (cv-v)

SLA NU DE BLADZIJDE MAAR OM, EN OMCIRKEL JE ANTWOORD
KORT HERHALEN VAN OPTIES

(PPNT-BEEN)
2) Zo ziet het **been** van een baby'tje eruit als het net geboren is. Waar lijkt het beentje van een baby het meest op als de baby nog een hele lange tijd in de buik van de moeder moet zitten?

a) Een beentje dat niet lijkt op het beentje van een pasgeboren baby. Het heeft een andere vorm (cv-v)
b) Een beentje dat er precies hetzelfde uitziet (cg-zonder groei)
c) Een beentje dat er precies hetzelfde uitziet maar dat wel kleiner is (cg- met groei)
d) Een beentje dat lijkt op het beentje van een geboren baby, maar er is alleen nog maar het bovenste stuk van het been (ci)

SLA NU DE BLADZIJDE MAAR OM, EN OMCIRKEL JE ANTWOORD.
KORT HERHALEN VAN OPTIES

(PPNT-OOR)
3) Zo ziet het **oor** van een baby eruit als deze geboren is. Waar lijkt het oor van een baby het meest op <u>als de baby nog een hele lange tijd in de buik van de moeder moet zitten?</u>
a) Het oor is er nog helemaal niet (ci)
b) Het oor ziet er precies hetzelfde uit maar het is wel kleiner (cg-met groei)
c) Het oor lijkt niet op het oor van een pasgeboren baby. Het heeft een andere vorm (cv-v)
d) Het oor ziet er precies hetzelfde uit (cg-zonder groei)

SLA NU DE BLADZIJDE MAAR OM, EN OMCIRKEL JE ANTWOORD.
KORT HERHALEN VAN OPTIES

(PPNT-VOET)
4) Zo ziet de **voet** van een baby'tje eruit als het net geboren is. Waar lijkt het voetje van een baby het meest op <u>als de baby nog een hele lange tijd in de buik van de moeder moet zitten?</u>
a) Een voetje dat er precies hetzelfde uitziet (cg-zonder groei)
b) Een voetje dat niet lijkt op de voet van een pasgeboren baby. Het heeft een andere vorm (cv-v)
c) Een voetje dat er precies hetzelfde uitziet maar dat wel kleiner is (cg-met groei)
d) Een voetje dat lijkt op het voetje van een geboren baby, maar het heeft nog niet alle tenen (ci)

SLA NU DE BLADZIJDE MAAR OM, EN OMCIRKEL JE ANTWOORD.
KORT HERHALEN VAN OPTIES

(PPNT-ARM)
5) Zo ziet het **armpje** van een baby'tje eruit als het net geboren is. Waar lijkt het armpje van een baby het meest op <u>als de baby nog een hele lange tijd in de buik van de moeder moet zitten?</u>
a) Een armpje dat lijkt op het armpje van een geboren baby, maar er is alleen nog maar het bovenste stuk van het armpje (ci)
b) Een armpje dat er precies hetzelfde uitziet maar dat wel kleiner is (cg- met groei)
c) Een armpje dat er precies hetzelfde uitziet (cg-zonder groei)
d) Een armpje dat niet lijkt op de arm van een pasgeboren baby. Het heeft een andere vorm (cv-v)

SLA NU DE BLADZIJDE MAAR OM, EN OMCIRKEL JE ANTWOORD.
KORT HERHALEN VAN OPTIES

(PPNT-OOG)

6) Zo zien het **oog** van een baby eruit als deze geboren is. Waar lijkt het oog van een baby het meest op als de baby nog een hele lange tijd in de buik van de moeder moet zitten?

a) Het oog lijkt niet op het oog van een pasgeboren baby. Het heeft een andere vorm (cv-v)
b) Het oog ziet er precies hetzelfde uit (cg-zonder groei)
c) Het oog ziet er precies hetzelfde uit maar het is wel kleiner (cg-met groei)
d) Het oog is er nog helemaal niet (ci)

SLA NU DE BLADZIJDE MAAR OM, EN OMCIRKEL JE ANTWOORD.
KORT HERHALEN VAN OPTIES

(PPNT-DRINK)
7) Als een baby geboren is krijgt het melk van de moeder die de baby met de mond opdrinkt. Zo krijgt de baby **drinken**. Hoe krijgt een baby drinken als de baby nog een hele lange tijd in de buik van de moeder moet zitten?

a) De baby in de buik krijgt ook drinken door zijn mond net zoals een geboren baby (cg)
b) De baby in de buik hoeft niet te drinken (ci)
c) De baby in de buik krijgt drinken via een soort buisje dat aan de navel vastzit (cv-f)

SLA NU DE BLADZIJDE MAAR OM, EN OMCIRKEL JE ANTWOORD.
KORT HERHALEN VAN OPTIES

(PPNT-WARM)
8) Als een baby geboren is, heeft het kleren aan om het **warm te hebben**. Hoe blijft een baby warm als de baby nog een hele lange tijd in de buik van de moeder moet zitten?

a) De baby in de buik hoeft het niet warm te hebben (ci)
b) De baby in de buik heeft het warm doordat de buik van de moeder het warm houdt (cv-f)
c) De baby in de buik heeft dan ook al kleren aan net zoals een geboren baby (cg)

SLA NU DE BLADZIJDE MAAR OM, EN OMCIRKEL JE ANTWOORD.
KORT HERHALEN VAN OPTIES

(PPNT-LUCHT)
9) Als een baby geboren is moet het **lucht inademen** om in leven te blijven. Hoe ademt een baby als de baby nog een hele lange tijd in de buik van de moeder moet zitten?

a) De baby in de buik krijgt wat van de lucht via het bloed van de moeder (cv-f)
b) De baby in de buik ademt ook lucht in net zoals een geboren baby (cg)
c) De baby in de buik heeft geen lucht nodig (ci)

SLA NU DE BLADZIJDE MAAR OM, EN OMCIRKEL JE ANTWOORD.
KORT HERHALEN VAN OPTIES

Late fase:
Nu stel ik jullie een aantal vragen over de baby in de buik van de moeder, vlàk voor dat deze geboren wordt. Hij heeft dan al best lang in de buik gezeten en wordt bijna geboren.

(PPNT-HAND)
10) Zo ziet de **hand** van een baby'tje eruit als het net geboren is. Waar lijkt het handje van een baby het meest op vlak voordat hij geboren wordt?

a) Een handje dat niet lijkt op het handje van een pasgeboren baby. Het heeft een andere vorm (cv-v)
b) Een handje dat er precies hetzelfde uitziet (cg-zonder groei)
c) Een handje dat er precies hetzelfde uitziet maar dat wel kleiner is (cg-met groei)
d) Een handje dat lijkt op het handje van een geboren baby, maar het heeft nog niet alle vingers (ci)

SLA NU DE BLADZIJDE MAAR OM, EN OMCIRKEL JE ANTWOORD.
KORT HERHALEN VAN OPTIES

(PPNT-BEEN)
11) Zo ziet het **been** van een baby'tje eruit als het net geboren is. Waar lijkt het beentje van een baby het meest op <u>vlak voordat hij geboren wordt?</u>
a) Een beentje dat lijkt op het beentje van een geboren baby, maar er is alleen nog maar het bovenste stuk van het been (ci)
b) Een beentje dat niet lijkt op het beentje van een pasgeboren baby. Het heeft een andere vorm (cv-v)
c) Een beentje dat er precies hetzelfde uitziet (cg-zonder groei)
d) Een beentje dat er precies hetzelfde uitziet maar dat wel kleiner is (cg- met groei)

SLA NU DE BLADZIJDE MAAR OM, EN OMCIRKEL JE ANTWOORD.
KORT HERHALEN VAN OPTIES

(PPNT-OOR)
12) Zo ziet het **oor** van een baby eruit als deze geboren is. Waar lijkt het oor van een baby het meest op <u>vlak voordat hij geboren wordt?</u>
a) Het oor ziet er precies hetzelfde uit (cg-zonder groei)
b) Het oor is er nog helemaal niet (ci)
c) Het oor ziet er precies hetzelfde uit maar het is wel kleiner (cg-met groei)
d) Het oor lijkt niet op het oor van een pasgeboren baby. Het heeft een andere vorm (cv-v)

SLA NU DE BLADZIJDE MAAR OM, EN OMCIRKEL JE ANTWOORD.
KORT HERHALEN VAN OPTIES

(PPNT-DRINK)
13) Als een baby geboren is krijgt het melk van de moeder die de baby met de mond opdrinkt. Zo krijgt de baby **drinken**. Hoe krijgt een baby drinken <u>vlak voordat hij geboren wordt?</u>
a) De baby in de buik krijgt drinken via een soort buisje dat aan de navel vastzit (cv-f)
b) De baby in de buik krijgt ook drinken door zijn mond net zoals een geboren baby (cg)
c) De baby in de buik hoeft niet te drinken (ci)

SLA NU DE BLADZIJDE MAAR OM, EN OMCIRKEL JE ANTWOORD.
KORT HERHALEN VAN OPTIES

(PPNT-WARM)
14) Als een baby geboren is, heeft het kleren aan om het **warm te hebben**. Hoe blijft een baby warm <u>vlak voordat hij geboren wordt?</u>
a) De baby in de buik heeft dan ook al kleren aan net zoals een geboren baby (cg)

b) De baby in de buik hoeft het niet warm te hebben (ci)
c) De baby in de buik heeft het warm doordat de buik van de moeder het warm houdt (cv-f)

SLA NU DE BLADZIJDE MAAR OM, EN OMCIRKEL JE ANTWOORD.
KORT HERHALEN VAN OPTIES

(PPNT-LUCHT)
15) Als een baby geboren is moet het **lucht inademen** om in leven te blijven. Hoe ademt een baby vlak voordat hij geboren wordt?

a) De baby in de buik heeft geen lucht nodig (ci)
b) De baby in de buik krijgt wat van de lucht via het bloed van de moeder (cv-f)
c) De baby in de buik ademt ook lucht in net zoals een geboren baby (cg)

SLA NU DE BLADZIJDE MAAR OM, EN OMCIRKEL JE ANTWOORD.
KORT HERHALEN VAN OPTIES

Dat was het dan.

(3) Nep-mens tekening

Nu gaan we het laatste deel doen. Jullie krijgen allemaal een leeg papier. Schrijf daarop je voornaam en je achternaam. Teken op het papier een gewoon mens. Ga je gang.

Na 3 minuten: ok, jullie mogen stoppen. Je mag nu je blaadje omdraaien. Teken nu op de achterkant van je papier een nep-mens. Dus niet een gewoon mens zoals jij zelf, maar een mens die niet bestaat. Misschien heb jij die mens zelf wel uitgevonden.

Na 10 minuten afsluiten en bedanken. Gummetjes/kleine cadeautjes uitdelen.

Vragenboekje

Naam: ________________________________

Leeftijd: ____

Klas: ____

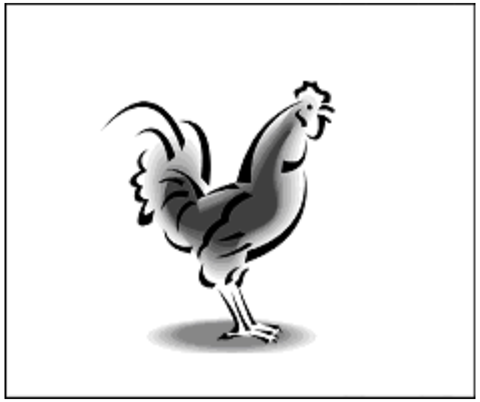 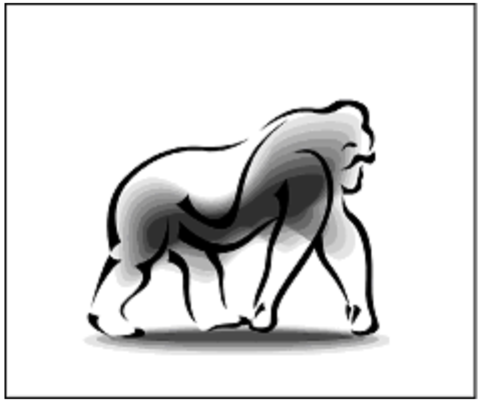 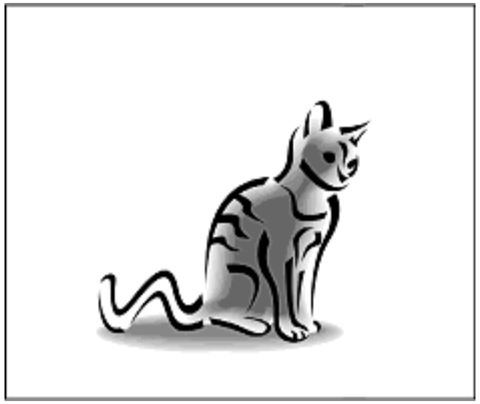

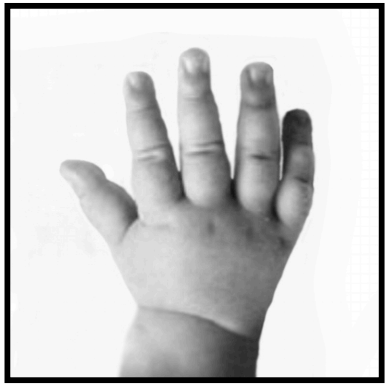

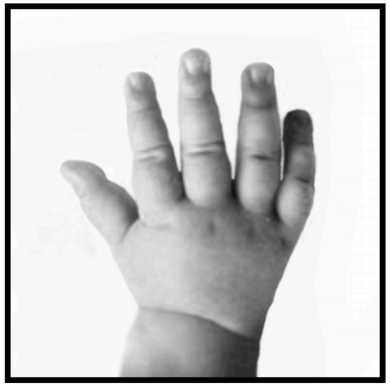

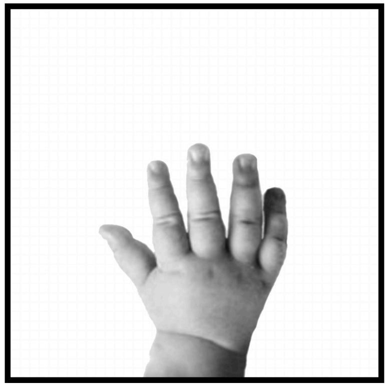

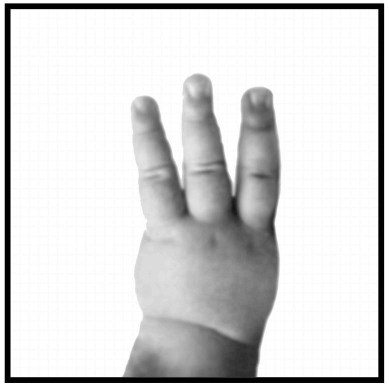

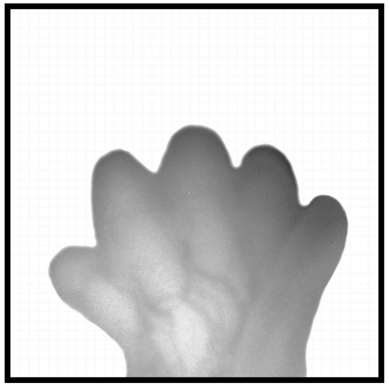

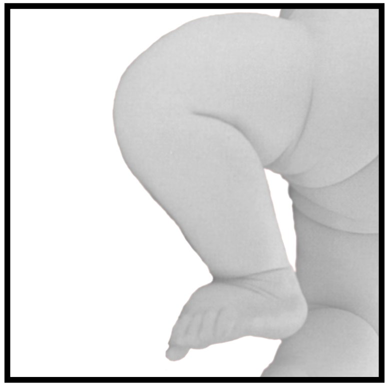

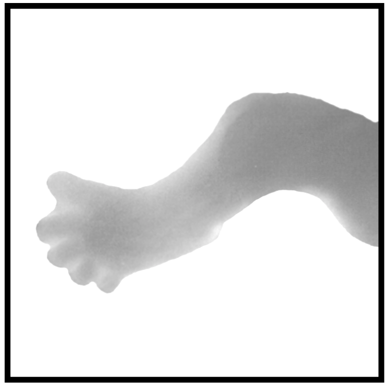

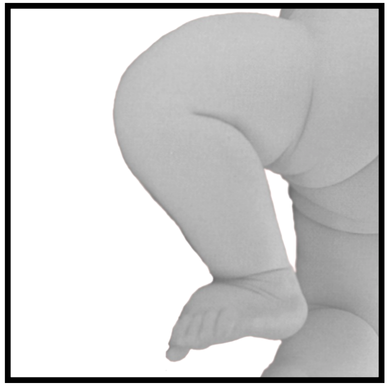

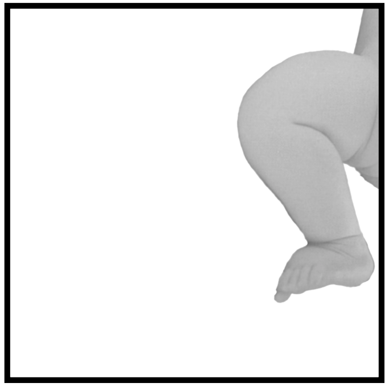

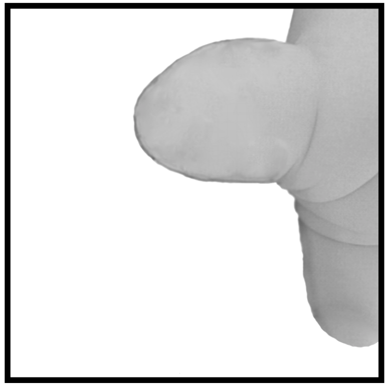

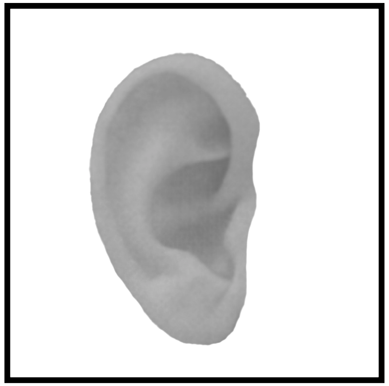

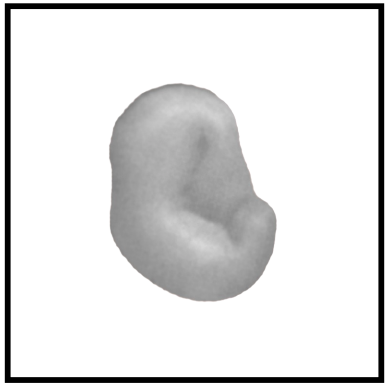

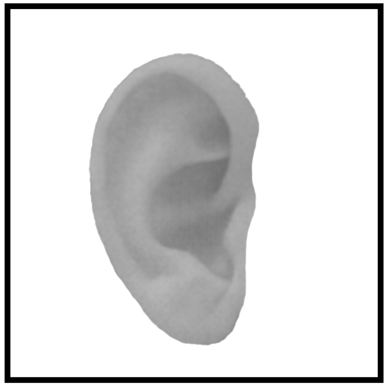

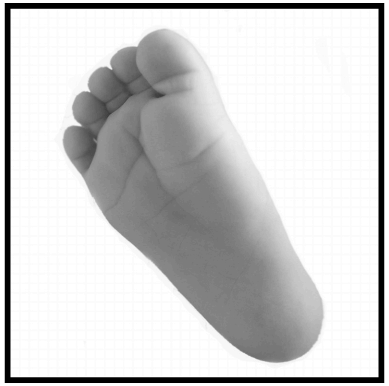

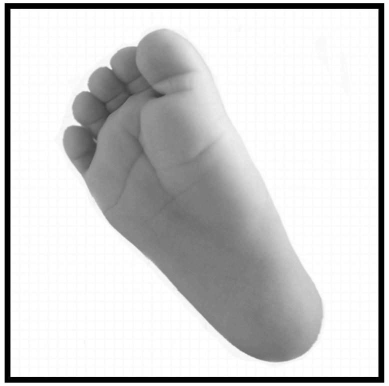

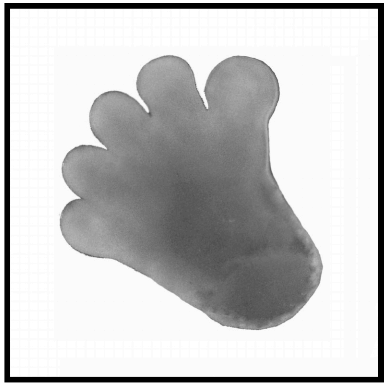

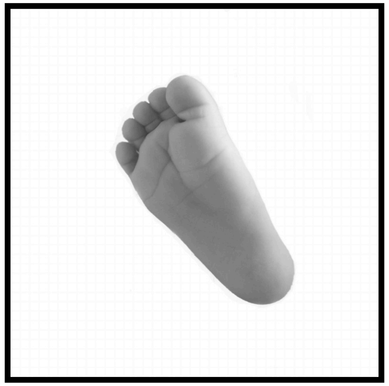

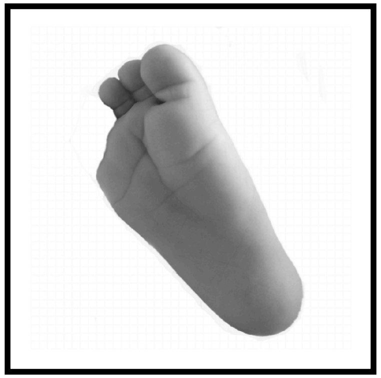

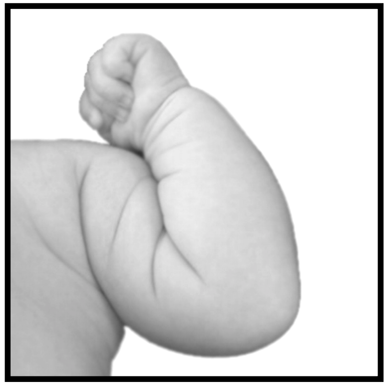

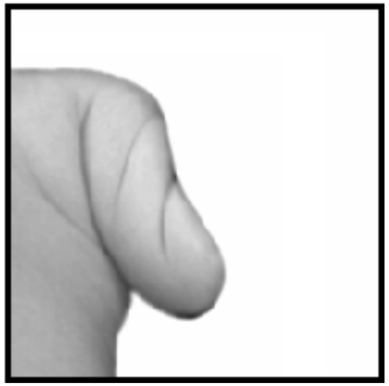

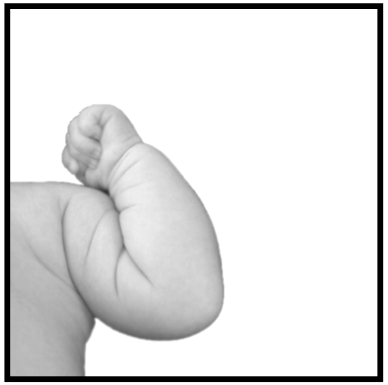

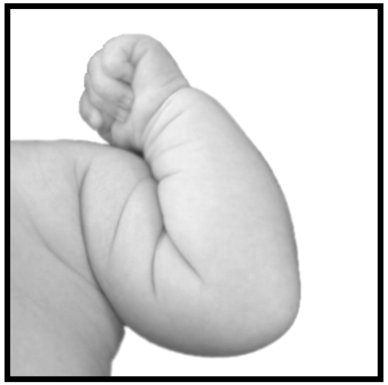

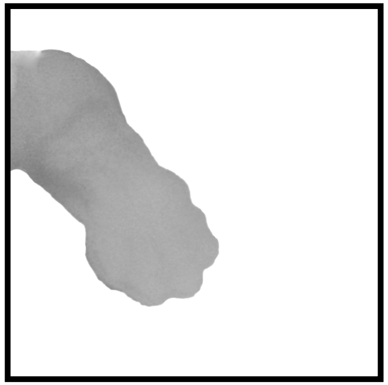

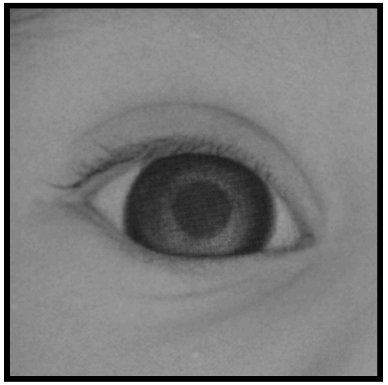

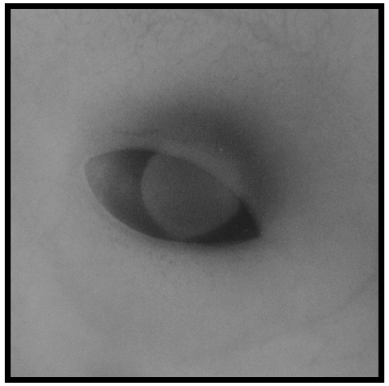
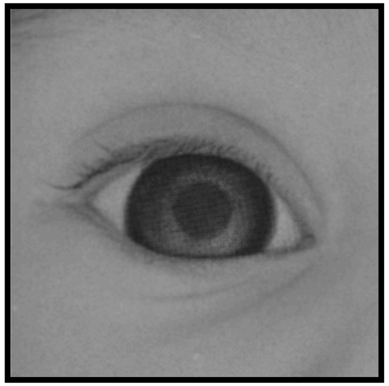
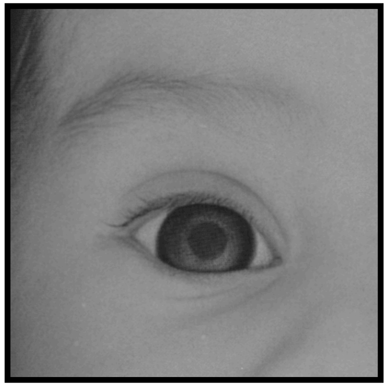
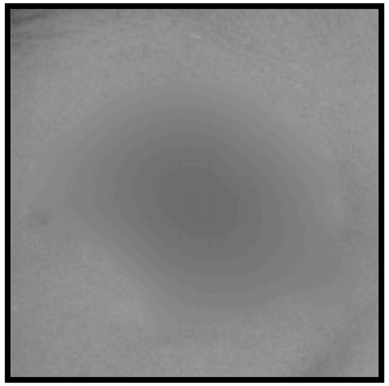

Drinken?

Melk door mond

Melk door mond	Niet	Buisje

Warm?

Kleren

Niet

Moeder
buik

Kleren

Lucht?
Ademen

Bloed
moeder

Ademen

Niet

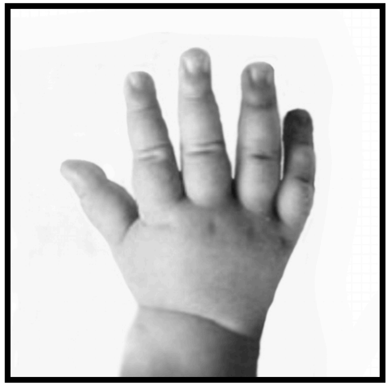

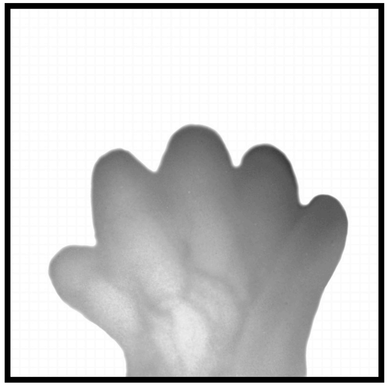 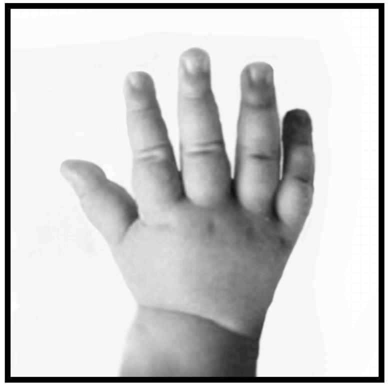 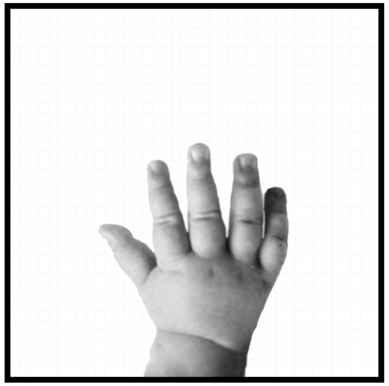 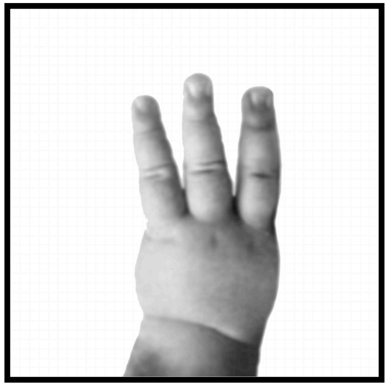

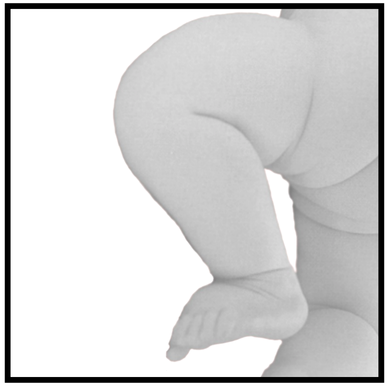

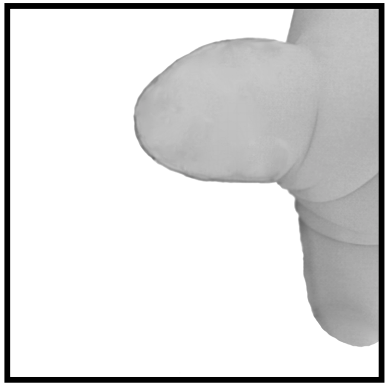
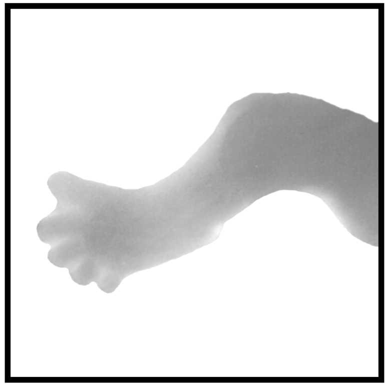
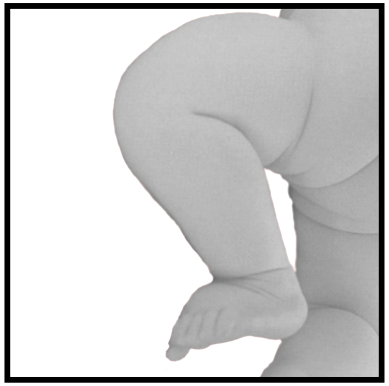
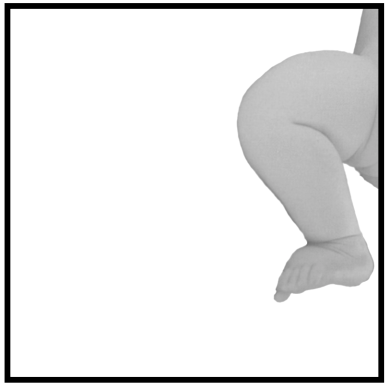

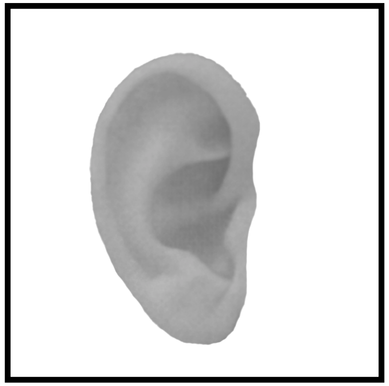

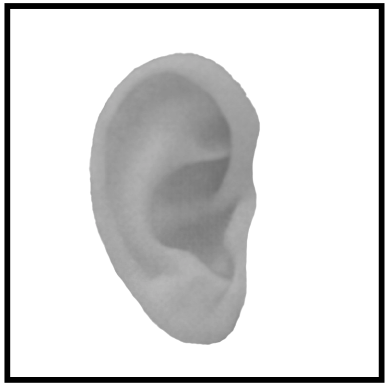

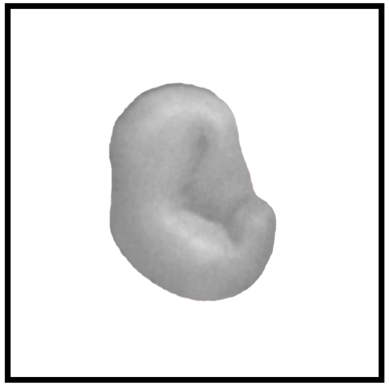

Drinken?

Melk door mond

Buisje

Melk door mond

Niet

Warm?
Kleren

Kleren

Niet

Moeder
buik

Lucht?
Ademen

Niet

Bloed
moeder

Ademen
